# Time to death and its predictors among adult patients on mechanical ventilation admitted to intensive care units in West Amhara comprehensive specialized hospitals, Ethiopia: a retrospective follow-up study

**DOI:** 10.1186/s12871-024-02495-9

**Published:** 2024-03-23

**Authors:** Kenubish Demem, Esubalew Tesfahun, Fetene Nigussie, Aster Tadesse Shibabaw, Temesgen Ayenew, Mengistu Abebe Messelu

**Affiliations:** 1 Nigist Eleni Comprehensive Specialized Hospital, Hosaena, Ethiopia; 2 Department of Public health, College of Medicine and Health Sciences, Debre Birhan University, Debre Birhan, Ethiopia; 3 Department of Nursing, College of Medicine and Health Sciences, Debre Birhan University, Debre Birhan, Ethiopia; 4 Department of Pediatrics and Child Health Nursing, College of Medicine and Health Sciences, Debre Markos University, Debre Markos, Ethiopia; 5 Department of Nursing, College of Medicine and Health Sciences, Debre Markos University, Debre Markos, Ethiopia

**Keywords:** Time to death, Predictors, Mechanical ventilation, Intensive care unit

## Abstract

**Introduction:**

Mechanical ventilation is the most common intervention for patients with respiratory failure in the intensive care unit. There is limited data from African countries, including Ethiopia on time to death and its predictors among patients on mechanical ventilators. Therefore, this study aimed to assess time to death and its predictors among adult patients on mechanical ventilation admitted in comprehensive specialized hospitals in West Amhara, Ethiopia.

**Methods:**

An institutional-based retrospective follow-up study was conducted from January 1, 2020, to December 31, 2022. A simple random sampling was used to select a total of 391 patients’ charts. Data were collected using data the extraction tool, entered into Epi-data version 4.6.0, and exported to STATA version 14 for analysis. Kaplan–Meier failure curve and the log-rank test were fitted to explore the survival difference among groups. The Cox regression model was fitted, and variables with a *p*-value < 0.25 in the bivariable Cox regression were candidates for the multivariable analysis. In the multivariable Cox proportional hazard regression, an adjusted hazard ratio with 95% confidence intervals were reported to declare the strength of association between mortality and predictors when a *p* value is < 0.05.

**Results:**

A total of 391 mechanically ventilated patients were followed for 4098 days at risk. The overall mortality of patients on mechanical ventilation admitted to the intensive care units was 62.2%, with a median time to death of 16 days (95% CI: 11, 22). Those patients who underwent tracheostomy procedure (AHR = 0.40, 95% CI: 0.20, 0.80), received cardio-pulmonary resuscitation (AHR = 8.78, 95% CI: 5.38, 14.35), being hypotensive (AHR = 2.96, 95% CI: 1.11, 7.87), and had a respiratory rate less than 12 (AHR = 2.74, 95% CI: 1.48, 5.07) were statistically significant predictors of time to death among mechanically ventilated patients.

**Conclusion:**

The mortality rate of patients on mechanical ventilation was found to be high and the time to death was short. Being cardiopulmonary resuscitated, hypotensive, and had lower respiratory rate were significant predictors of time to death, whereas patients who underwent tracheostomy was negatively associated with time to death. Tracheostomy is needed for patients who received longer mechanical ventilation, and healthcare providers should give a special attention for patients who are cardiopulmonary resuscitated, hypotensive, and have lower respiratory rate.

**Supplementary Information:**

The online version contains supplementary material available at 10.1186/s12871-024-02495-9.

## Introduction

Mechanical ventilation (MV) is a lifesaving treatment modality considered a cornerstone to support critically ill patients and is used most commonly in Intensive Care Units (ICU) [1–5]. Globally, the number of patients requiring mechanical ventilation is increasing, specifically patients with Acute Respiratory Failure (ARF). It is provided for over 20 million patients worldwide [6], and among ICU-admitted patients, 35–50% require MV during their admission [7]. Millions of patients are discharged from hospitals after surviving critical illnesses each year. However, patients with ARF had a low survival rate, with a death rate of 67.2%. As a result, these patients are often supported with MV to improve gas exchange, reduce the work of breathing, prevent complications, and improve their outcome [8]. On the contrary, due to complications resulting from mechanical ventilation, up to 40% of patients died in the hospital because of its harmful effect on the patients’ lungs [9].

Despite the advancement in technology to support medical practices in the ICU, the death rate among mechanically ventilated patients remains high [2]. This mortality might be due to factors existed prior to the initiation of MV or developed during the course of treatment, as well as its complications [10]. Moreover, survivors of mechanically ventilated critically ill patients had short-term survival and a poor quality of life [11]. In addition to the high mortality rate, mechanically ventilated patients had faced various health-related problems [12, 13], such as emotional signs when they are stressed, mental health problems [14], physiological disturbances, and physical changes [15]. Besides, these critically ill patients on MV had increased healthcare expenditures since these patients consume extensive medical resources [16, 17], such as medical equipment, supplies, and personnel costs required to provide care [18].

In Ethiopia, many studies have been conducted to determine the mortality outcome of patients admitted to the ICU. However, studies on the survival of patients on mechanical ventilation admitted to the ICU were scarce. Furthermore, even available studies are not specific and cross-sectional in nature, which couldn’t consider the time variable and other clinically significant variables. In this regard, evidence about the time to death and its predictors helps to improve patient outcomes, assist clinicians in designing evidence-based strategies, decision-making process, and better resource allocation, and is used as an input for future studies. Therefore, this study aimed to determine the time to death and its predictors among mechanically ventilated patients admitted to the ICU.

## Methods and materials

### Study area

The study was conducted in West Amhara comprehensive specialized hospitals. There are five comprehensive specialized hospitals found in West Amhara. Among these, Felege-Hiwot, Tibebe Ghion, and Debre Markos comprehensive specialized hospitals were included in the study. Felege-Hiwot and Tibebe Gion comprehensive specialized hospitals are found in Bahir Dar, which is located 565 km northwest of Addis Ababa, the capital city of Ethiopia, whereas Debre Markos comprehensive specialized hospital is found in Debre Markos town, 299 km away from Addis Ababa. These hospitals provide inpatient and outpatient healthcare services for more than 15 million people. The ICUs are providing similar level of care equipped with mechanical ventilators, noninvasive hemodynamic monitoring devices, portable ultrasounds, Electrocardiograms (ECG), defibrillators, and infusion pumps. The total number of ICU beds in Felege Hiwot, Tibebe Ghion, and Debre Markos is 12, 9, and 4, respectively.

### Study design and period

The institutional-based retrospective follow-up study design was conducted from January 1, 2020, to December 31, 2022, and the actual data collection period was December 1–31, 2022.

### Population

All adult patients who were admitted to the ICU in West Amhara comprehensive specialized hospitals and received MV were the source population. All adult patients on MV who were admitted to the ICU in selected comprehensive specialized hospitals during the study period were the study population. Records of patients on MV during the study period and whose age ≥ 18 years were included. However, records of patients on MV without the outcome variable were excluded from the study.

#### Sample size determination

A survival sample size calculation power approach using Stata 14 software with cox proportional assumptions was used.

$$N = \frac{E}{{P(E)}}\,where\,E = \frac{{{{\left( {\frac{{z\alpha }}{2} + Z\beta } \right)}^2}}}{{\rho (1 - \rho )Ln{{(HR)}^2}}}$$

Where: N = total number of sample size, E = total number of events required, p(E) = probability of event,ρ**=**proportion of subjects under exposure variable,*HR*=hazard ratio,$\frac{{z\alpha }}{2}$ =critical value at 95% confidence level, which 1.96 and $Z\beta$= 0.842, standard deviation and correlation of covariate 0.5 and 0, respectively, and 0.1 for probability of withdrawal.

Therefore, the sample size was calculated for the four predictor variables, i.e., use of vasopressors, neuromuscular blockers, hemodialysis, and body mass index ≤ 21 kg/m^2^ based on the study done in Korea [11] (Table 1).

**Table 1:** Sample size calculation to assess time to death and its predictors among mechanically ventilated patients in West Amhara comprehensive specialized hospitals, Ethiopia, 2022

Assumption	Variable	P(E)	Hazard ratio	Chart attrition rate	Sample size
Power = 80% Z$a$_/2_=1.96 (α) = 0.05 P(w) = 0.1	Neuromuscular blockers	0.568	2.43	10%	150
	Use of vasopressors	0.294	2.31		325
	Hemodialysis	0.524	1.91		305
	Body mass index ≤ 21 kg/m^2^	0.516	1.83		355

Accordingly, the largest sample size obtained was 355. Thus, by adding 10% for possible incomplete charts and lost medical records, the total sample size was **391.**

### Sampling techniques and procedures

**Fig. 1:**
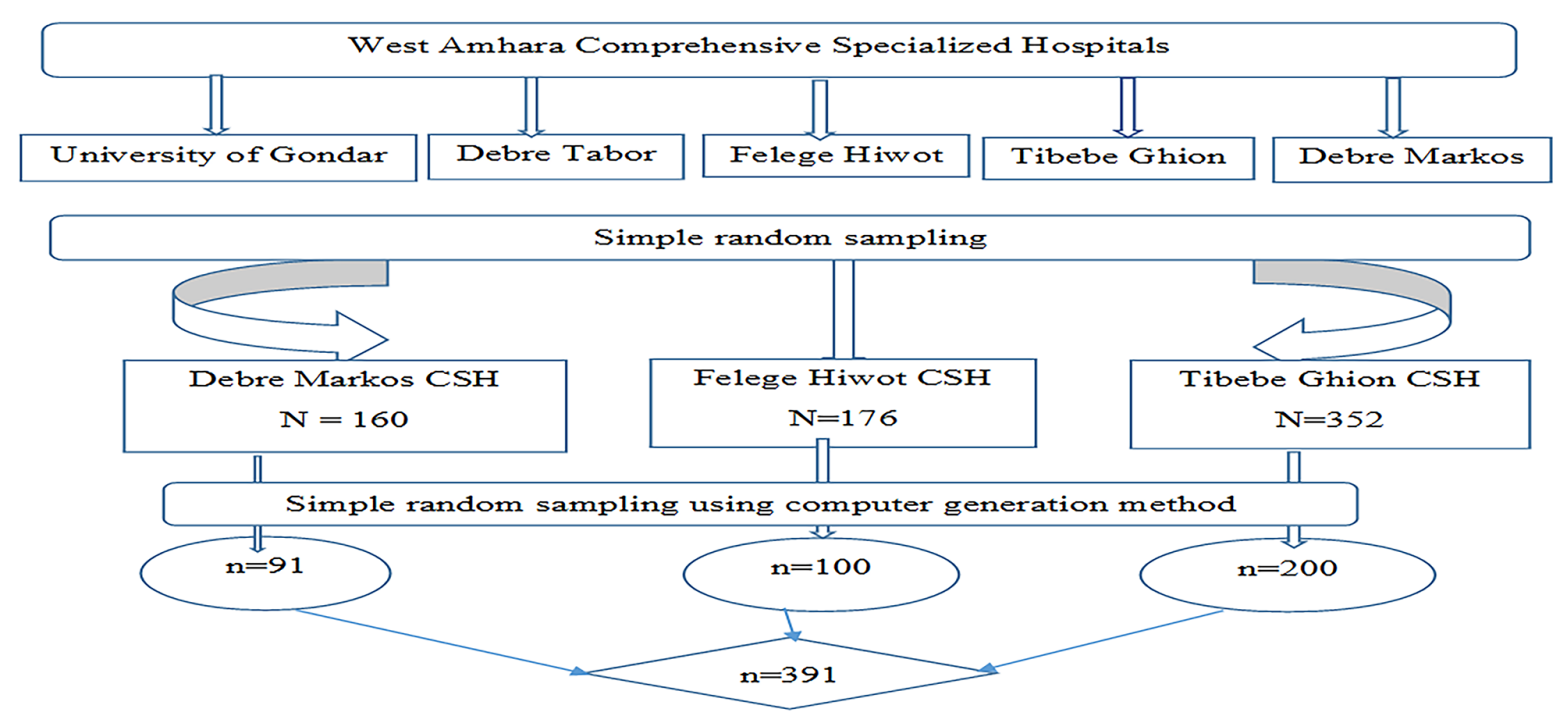
Schematic presentation of sampling procedure to select mechanically ventilated patients admitted to the ICU in comprehensive specialized hospitals in West Amhara, 2022. NB: CSH (Comprehensive Specialized Hospital)

A simple random sampling was employed to select three hospitals from a total of five comprehensive specialized hospitals. Moreover, after the sample size was proportionally allocated for each hospital, a simple random sampling technique using the computer generation method was used to select the patients’ charts. The list of patients who were admitted to the ICU and received MV was used as a sampling frame. The total number of patients who received MV and were admitted to the ICU in two years was 688 (Fig. 1).

### Operational definitions

#### Prolonged use of mechanical ventilation

when patients experience difficulty of weaning from MV after 21 days (the use of mechanical ventilation for ≥ 21 days) [19].

#### Incomplete card

patient chart in which the outcome of the patient and the duration of mechanical ventilation were not registered.

#### Comorbidity

presence of disease conditions according to the Carlson comorbidity index. A “yes” to these disease conditions indicates comorbidity, and “no” answer was taken as the absence of comorbidity [20].

#### Event

mechanically ventilated adult patients who died in the hospital during the follow-up period.

#### Censored

patients who did not develop the outcome of interest (death) such as Left Against Medical Advice (LAMA), transferred out, and recovered during the follow-up period.

#### Time to death

The number of days starting from the initiation of the mechanical ventilator to the occurrence of an event (death) within the follow-up period.

### Data collection tool and procedures

Data was collected using a data extraction tool adapted from different literature [2, 4, 11, 21, 22]. The questionnaire has five sections: factors related to socio-demographic characteristics, factors present at the initiation of mechanical ventilation, factors related to the patient’s clinical condition, factors related to the management of patients, and factors related to vital signs at admission (Supplementary material 1). The data were obtained from the ICU registration book and patients’ charts. Data collection was done by four BSc nurses, and one MSc nurse supervised the collection period process.

### Data quality control

A one-day training was provided to data collectors and supervisor on the data collection process. Preliminary chart review was conducted in Debre Markos comprehensive specialized hospital to ensure the availability of variables on the patient’s chart. Moreover, the data were cross-checked for consistency, completeness, clarity, and accuracy by the supervisor and the principal investigator before analysis.

### Data processing and analysis

Data were entered into Epi Data Version 4.6 and exported into Stata Version 14 statistical software for analysis. Descriptive statistics of numeric variables are presented using the median with Inter Quartile Range (IQR) and categorical variables expressed in frequency with percentages. The incomplete data were managed with the assumption of multiple imputations, after which it was ascertained that the missing data was completely at random and less than 15% of the records. The outcome of each participant was dichotomized into censored or event. The Kaplan-Meier failure curve was used to estimate failure time. The Kaplan-Meier failure curve and the log-rank test were fitted to test the presence of differences between different categories of explanatory variables. The proportional hazard assumption was checked both graphically and statistically by using the log (-log) plot and Schoenfeld residual test, respectively, and it was satisfied. Multicollinearity for independent variables was checked by using Variance Inflation Factor (VIF) and the mean VIF was 1.37. The Cox proportional hazard regression was used to explore the association between each independent variable and the outcome variable. The model’s fitness was tested using the Cox–Snell residual test. Variables having a *p*-value < 0.25 in the bivariable analysis were candidates for the multivariable analysis. An Adjusted Hazard Ratio (AHR) with 95% Confidence Intervals (CI) was computed to explore the strength of the association, and variables with a *p*-value less than 0.05 were considered statistically significant with time to death.

## Result

### Socio-demographic characteristics of the study participants

During the study period, 688 patients were treated with invasive mechanical ventilation. From these, 391 patients’ charts were reviewed (Supplementary material 2). About 159 (40.7%) of the participants were less than 45 years old, with a median age of 50 years (IQR: 18–90). Nearly two-thirds (63.4%) were males, and more than half (54.5%) were rural residents (Table 2).

**Table 2:** Socio-demographic characteristics of patients on MV admitted to ICU in West Amhara Comprehensive specialized hospitals, Ethiopia, 2022

Variables	Categories	Death(*N* = 243)	Censored(*N* = 148)	Total (%)	IDR
Age in years	≤ 44	87	72	159 (40.7%)	0.069
	45–54	37	26	63 (16.1%)	0.053
	55–64	55	18	73 (18.7%)	0.067
	65–74	45	18	63 (16.1%)	0.055
	≥ 75	19	14	23 (5.9%)	0.037
Gender	Male	157	91	248 (63.4%)	0.057
	Female	86	57	143 (36.6%)	0.063
Residence	Urban	115	63	178 (45.5%)	0.060
	Rural	128	85	213 (54.5%)	0.058

### Vital signs of mechanically ventilated patients during admission

Nearly half (45.5%) and three-fourths (73.40%) of study participants were hypotensive and hypoxic during admission, respectively (Table 3).

**Table 3:** Vital signs during admission among mechanically ventilated patients admitted to ICU in West Amhara Comprehensive specialized hospitals, Ethiopia, 2022

Variables	Categories	Death(*N* = 243)	Censored(*N* = 148)	Total (%)	IDR
Systolic blood pressure/mmHg	< 90	146	32	178 (45.5%)	0.115
	90–140	36	20	56 (14.3%)	0.046
	> 140	61	96	157 (40.2%)	0.058
Diastolic blood pressure/mmHg	< 60	45	19	64(16.4%)	0.086
	60–90	165	103	268(68.5%)	0.044
	> 90	33	26	59(15.1%)	0.058
Heart rate/min	< 60	10	8	18(4.6%)	0.264
	60–100	96	69	165(42.2%)	0.054
	> 100	133	75	208(53.2%)	0.059
Respiratory rate/min	< 12	131	98	229 (58.5%)	0.049
	12–20	50	10	60 (15.4%)	0.044
	> 20	62	40	102 (26.1%)	0.071
Oxygen saturation	< 90%	182	105	287(73.4%)	0.060
	≥ 90%	61	43	104(26.6%)	0.056
Temperature in ℃	< 35.5	27	21	48(12.3%)	0.058
	35.5–38.5	172	112	284(72.6%)	0.053
	> 38.5	44	15	59(15.1%)	0.088

### Clinical-related characteristics of the study participants

More than a quarter (28.6%) of patients were admitted to the ICU with the diagnosis of respiratory failure, followed by neurological disorder (16.4%). Nearly half (48.3) of the study participants had a Glasgow Coma Scale (GCS) score of 3–8 (Table 4).

**Table 4:** Clinical-related characteristics of patients on MV admitted to ICU in West Amhara Comprehensive specialized hospitals, Ethiopia, 2022

Variables	Categories	Death (*N* = 243)	Censored (*N* = 148)	Total (%)	IDR
Diagnosis during ICU admission	ARF	69	43	112 (28.6%)	0.068
	Neuromuscular	12	11	23 (5.9%)	0.056
	Cardiovascular	40	17	57 (14.6%)	0.063
	Neurological	33	31	64 (16.4%)	0.079
	Gastrointestinal	13	14	27 (6.9%)	0.066
	Others	69	39	108 (27.6%)	0.049
GCS at admission	3–8	115	74	189 (48.34)	0.064
	9–12	31	20	51 (13.04)	0.057
	13–15	97	54	151(38.62)	0.054
Patient type	Medical	182	94	276(70.59%)	0.062
	Surgical	36	28	64 (16.37%)	0.047
	Obstetrics	11	15	26 (6.65%)	0.055
	Emergency	14	11	25 (6.39%)	0.068
Admission source	Emergency	92	55	147 (37.6%)	0.067
	Ward	65	34	99 (25.3%)	0.061
	Operating room	11	11	22 (5.6%)	0.029
	Referred	75	48	123 (31.5%)	0.058
Comorbidity	Yes	151	72	223 (57.0%)	0.065
	No	92	76	168 (43.0%)	0.056
Acute Respiratory Distress Syndrome	Yes	67	38	105 (26.6%)	0.074
	No	176	110	286 (73.4%)	0.055
Pneumonia	Yes	114	62	176 (45.0%)	0.077
	No	129	86	215 (55.0%)	0.049

Others; renal, hematologic, hepatic problems

ARF; Acute Respiratory Failure, GCS; Glasgow Coma Scale

### Management-related characteristics of patients on MV

More than one-fourth (27.62%) of the study participants were received vasopressors during their ICU admission. Tracheostomy procedure is performed for only (8.4%) of the mechanically ventilated patients (Table 5).

**Table 5:** Management-related characteristics of patients on MV admitted to the ICU in West Amhara Comprehensive specialized hospitals, Ethiopia, 2022

Variables	Categories	Death(*N* = 243)	Censored(*N* = 148)	Total (%)	IDR
Vasopressors	Yes	97	11	108 (27.6%)	0.077
	No	146	137	283 (72.4%)	0.051
Neuromuscular blocker	Yes	37	19	56 (14.3%)	0.060
	No	206	129	335 (85.7%)	0.059
Tracheostomy	Yes	18	15	33 (8.4%)	0.035
	No	225	133	358 (91.6%)	0.067
Cardiopulmonary resuscitation	Yes	161	14	175 (44.8%)	0.114
	No	82	134	216 (55.2%)	0.029
Dialysis	Yes	17	10	27(6.9%)	0.051
	No	221	143	364(93.1%)	0.060

### Ventilation-related characteristics of the study participants

The majority of patients (90.0%) were received Synchronized Intermittent Mandatory Ventilation (SIMV) mode of mechanical ventilation. From the total mechanical ventilated patients, (15.5%) were developed ventilated associated complications (Table 6).

**Table 6:** Ventilation-related characteristics of the study participants of patients on MV admitted to ICU in West Amhara Comprehensive specialized hospitals, Ethiopia, 2022

Variables	Categories	Death(*N* = 243)	Censored(*N* = 148)	Total (%)	IDR
Reason for mechanical ventilation	Respiratory	147	90	237(60.6%)	0.067
	Neurologic	44	40	84(21.5%)	0.043
	Neuromuscular	22	13	35(9.0%)	0.048
	Others	23	12	35 (8.9%)	0.053
Mode	Assisted control	231	121	352(90.0%)	0.062
	SIMV	12	27	39(10.0%)	0.031
Ventilator associated complications	Yes	46	15	61(15.6%)	0.031
	No	197	133	330(84.4%)	0.074

Others; cardiovascular, poisoning and renal; SIMV = Synchronized Intermittent Mandatory Ventilation

### Assessing the proportional hazard assumption

The Cox proportional hazard assumptions were checked statistically and graphically using the global test and log (-log) plot, respectively. All the covariates met the proportional hazard assumptions, and the *p*-value overall Schoenfeld global test was 0.0795 (Table 7).

**Table 7:** Proportional hazard assumption test by Schoenfeld residuals for each predictor variable

Predictors	Rho	Chi^2^	Df	Prob > Chi^2^
Age	0.02903	0.14	1	0.7108
Diagnosis	0.01004	0.02	1	0.9002
GCS score	-0.01893	0.06	1	0.8060
Vasopressor	-0.11868	2.51	1	0.1134
Tracheostomy	-0.03377	0.21	1	0.6505
Pneumonia	-0.00008	0.00	1	0.9992
CPR	0.13534	2.99	1	0.0838
Heart rate	-0.00078	0.00	1	0.9914
SBP	0.03049	0.19	1	0.6620
Temperature	0.05503	0.61	1	0.4330
Respiratory rate	-0.01013	0.03	1	0.8707
DBP	-0.02505	0.11	1	0.7416
Comorbidity	0.02663	0.11	1	0.7378
Global test		21.96	13	0.0795

Df (Degree of freedom), Prob > chi2 (*p*-value), Rho (spearman correlation), SBD; Systolic Blood Pressure, DBP; Diastolic Blood Pressure

### Model goodness of fit test

**Fig. 2:**
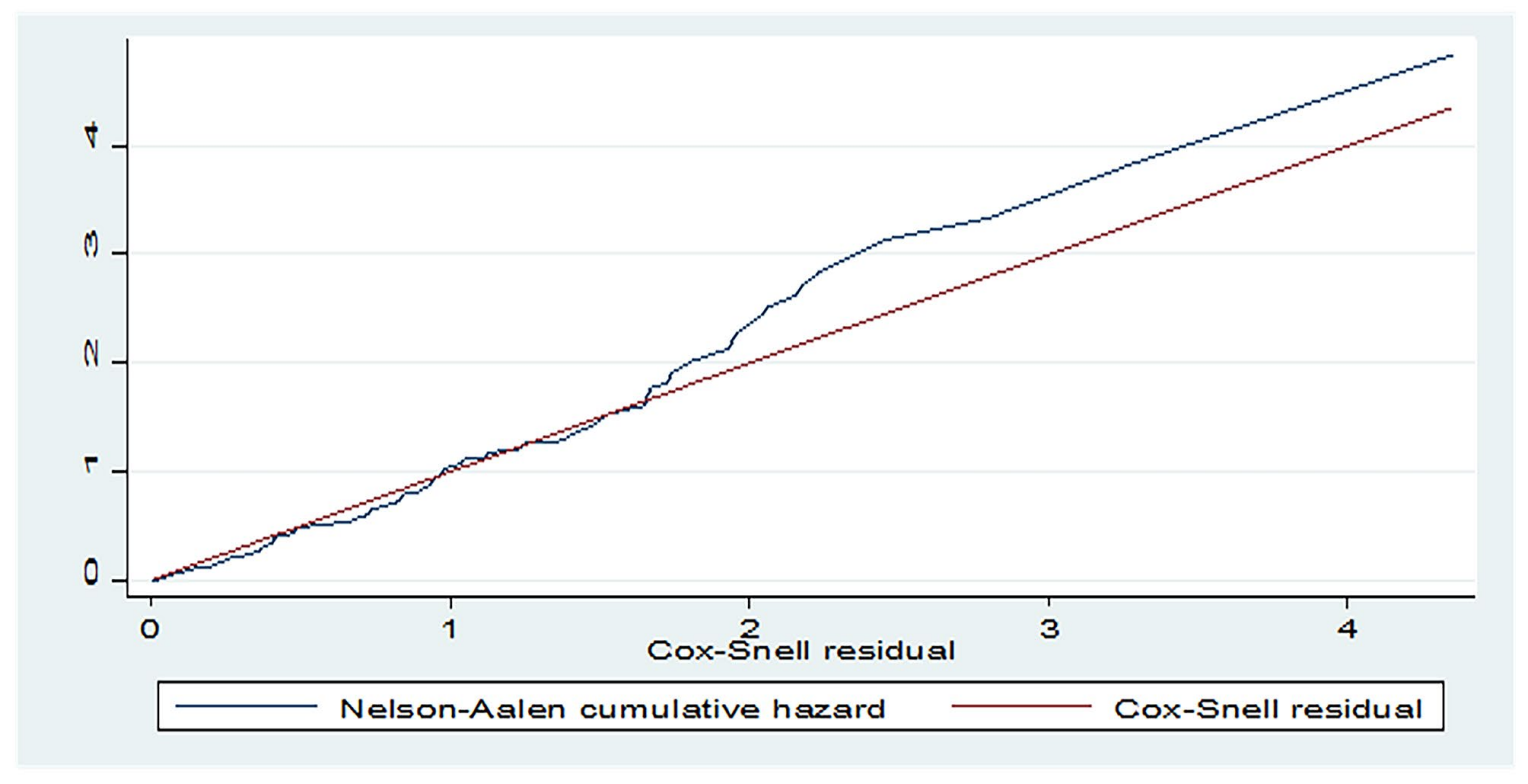
Nelson-Aalen cumulative hazard graph against Cox-Snell residual for patients on MV and admitted to the ICU in West Amhara comprehensive specialized hospitals, 2022

Cox Snell residuals were used to check the goodness of fit test. The hazard function follows 45˚ closest to the baseline hazard, which showed that the model was well fitted (Fig. 2).

### Time to death among mechanically ventilated patients

**Fig. 3:**
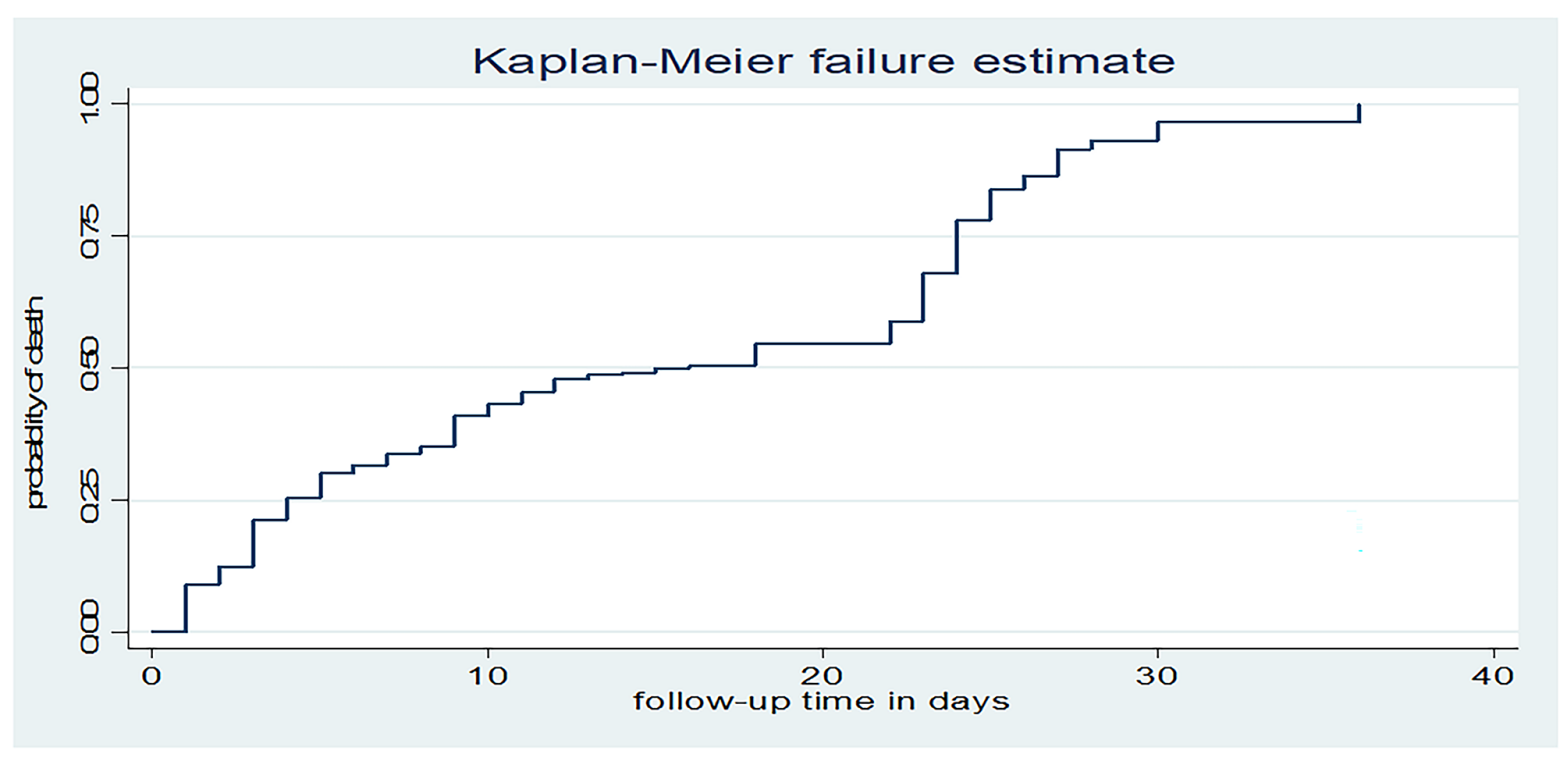
Kaplan-Meier failure function for patients on MV admitted to ICU in West Amhara comprehensive specialized hospitals: Ethiopia, 2022

This study found that the overall mortality of patients on mechanical ventilation admitted to the ICU was 62.2%, with a median time to death of 16 days (95% CI: 11, 22). Patients were followed for a minimum of 1 day and a maximum of 36 days, with a total person-time at risk of 4098 days. The overall incidence density rate of mortality among mechanically ventilated patients admitted to the ICU was 5.93 per 100 person-day observations (95% CI: 5.23, 6.72). According to the Kaplan-Meier failure curve, the cumulative probability of death at the end of 8, 16, 24, 32 and 36 days was 5.8%, 3.6%, 7.3%, 23%, and 25.0%, respectively (Fig. 3).

### Test for equality of failure function

**Fig. 4:**
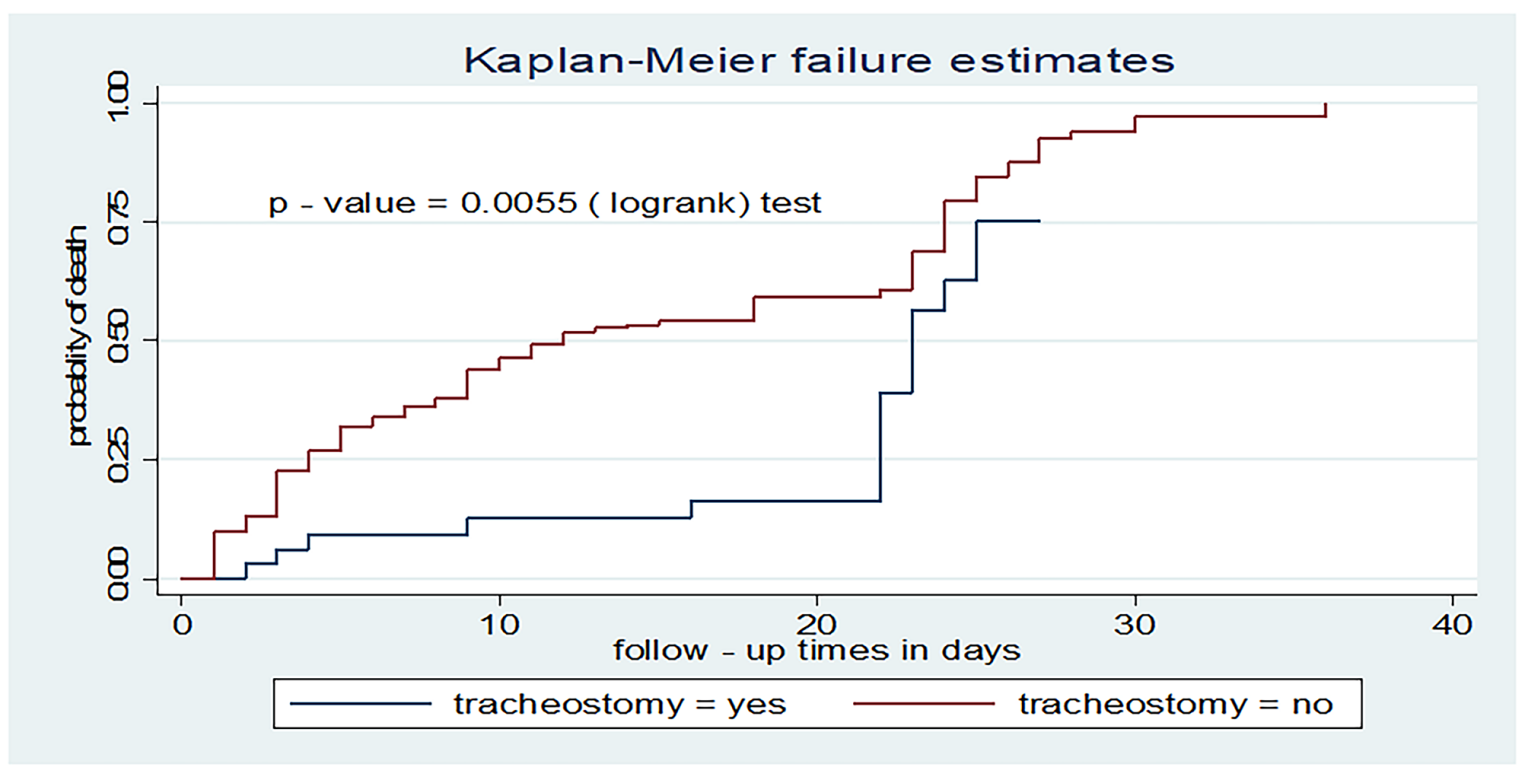
Kaplan-Meier failure curve for tracheostomy among adult patients on mechanical ventilation admitted to ICU comprehensive specialized hospitals in West Amhara, 2022

A Kaplan-Meier failure curve together with the log-rank test was done to test the presence of a difference in the time to death among predictor variables. According to the current study, those patients without a tracheostomy procedure had a shorter time to death with a median survival time of 12 days (95% CI: 10, 18) as compared to their counterparts (23 days, 95% CI: 22, 25) (Fig. 4).

**Fig. 5:**
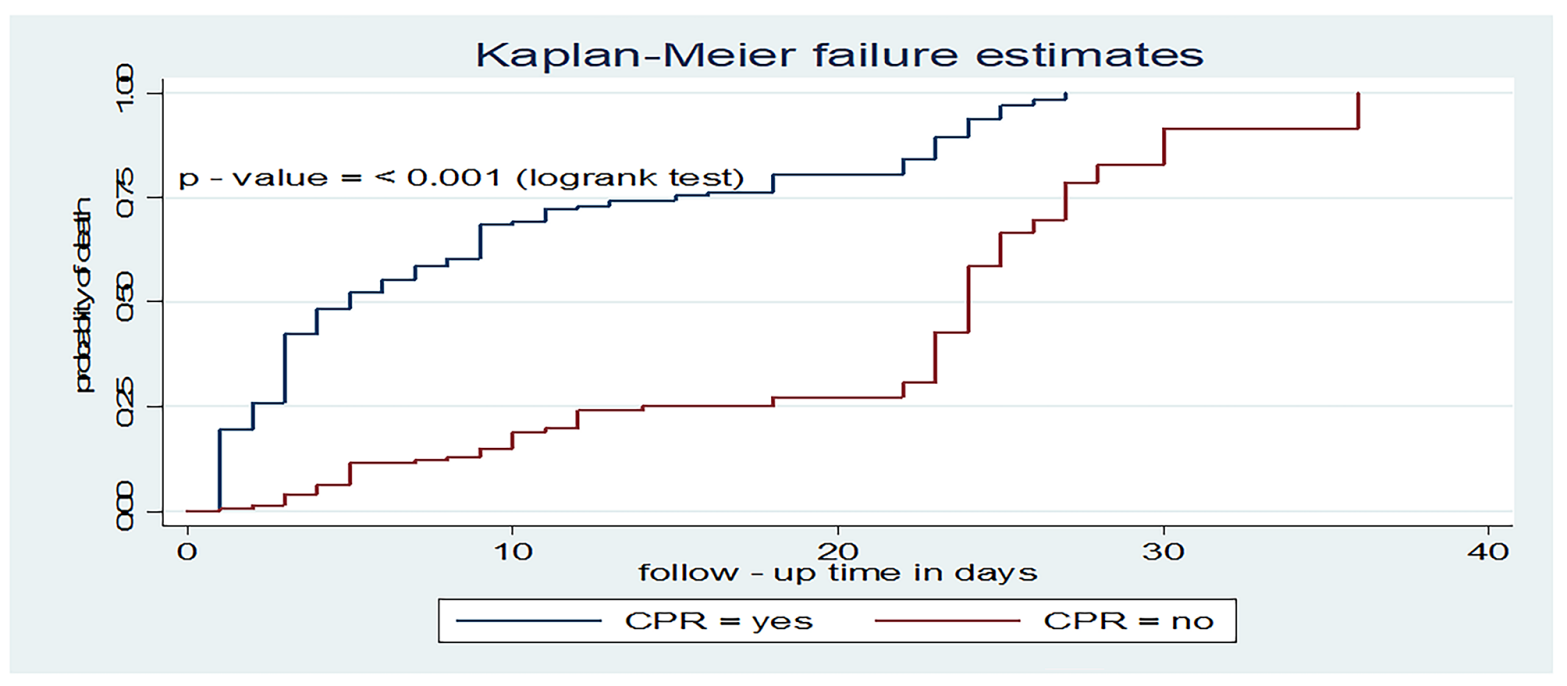
Kaplan-Meier failure curve for CPR among patients on MV admitted to the ICU in comprehensive specialized hospitals in West Amhara, 2022

In addition, those patients who received CPR had a shorter time to death with a median survival time of 5 days (95% CI: 3, 7) as compared to their counterparts (24 days, 95% CI: 23, 25) (Fig. 5).

**Fig. 6:**
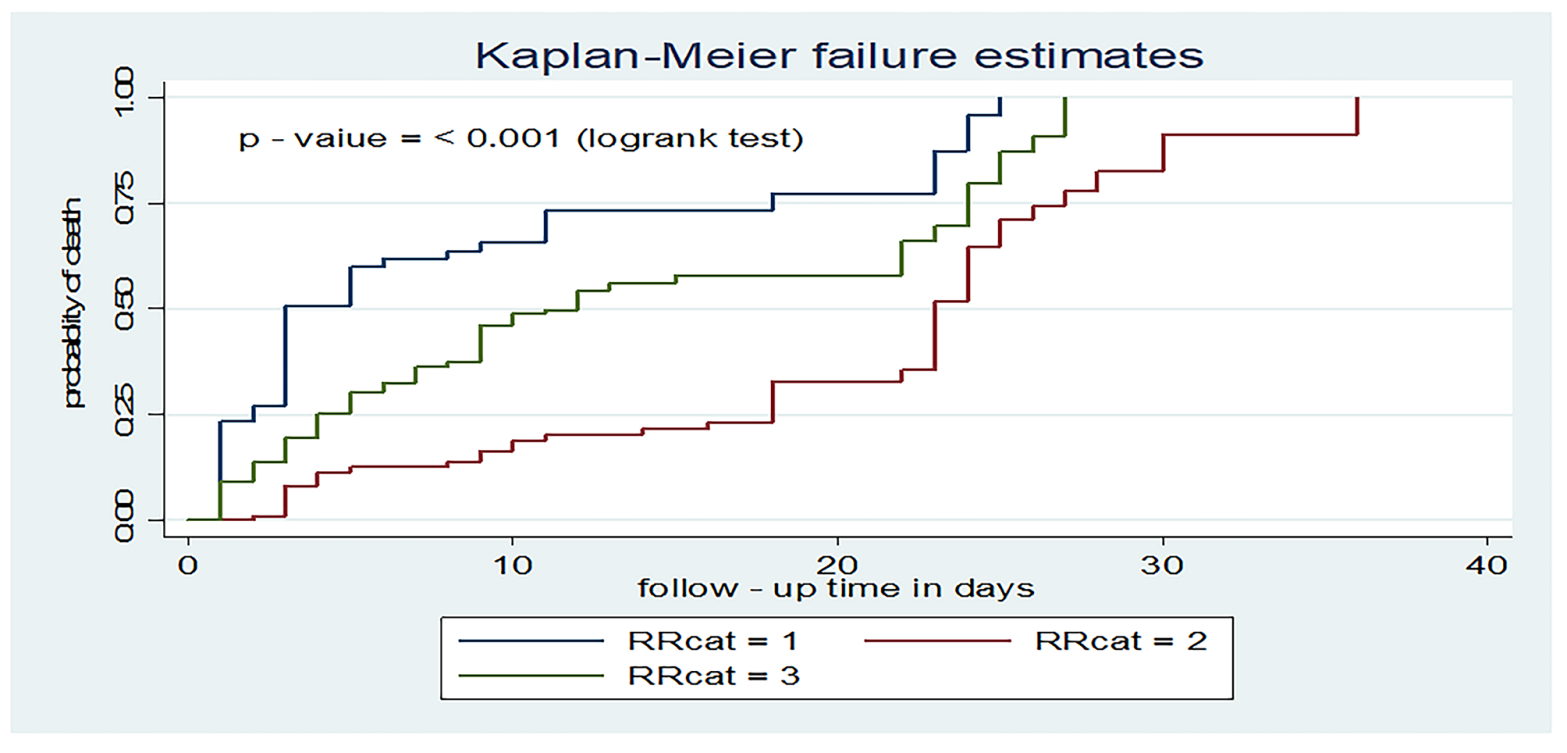
Kaplan-Meier failure curve for respiratory rate among adult patients on mechanical ventilation admitted to ICU comprehensive specialized hospitals in West Amhara, 2022

Patients who had a respiratory rate less than 12 had a shorter time to death with a median survival time of 3 days (95% CI: 3, 8), as compared to those who had 12–20 breaths/minutes (23 days, 95% CI: 23, 24). Similarly, those patients with a respiratory rate greater than 20 breaths/ min had a shorter time to death with a median survival time of 12 days (95% CI: 9, 15) compared to those who had a respiratory rate of 12–20 breaths/min (Fig. 6).

**Fig. 7:**
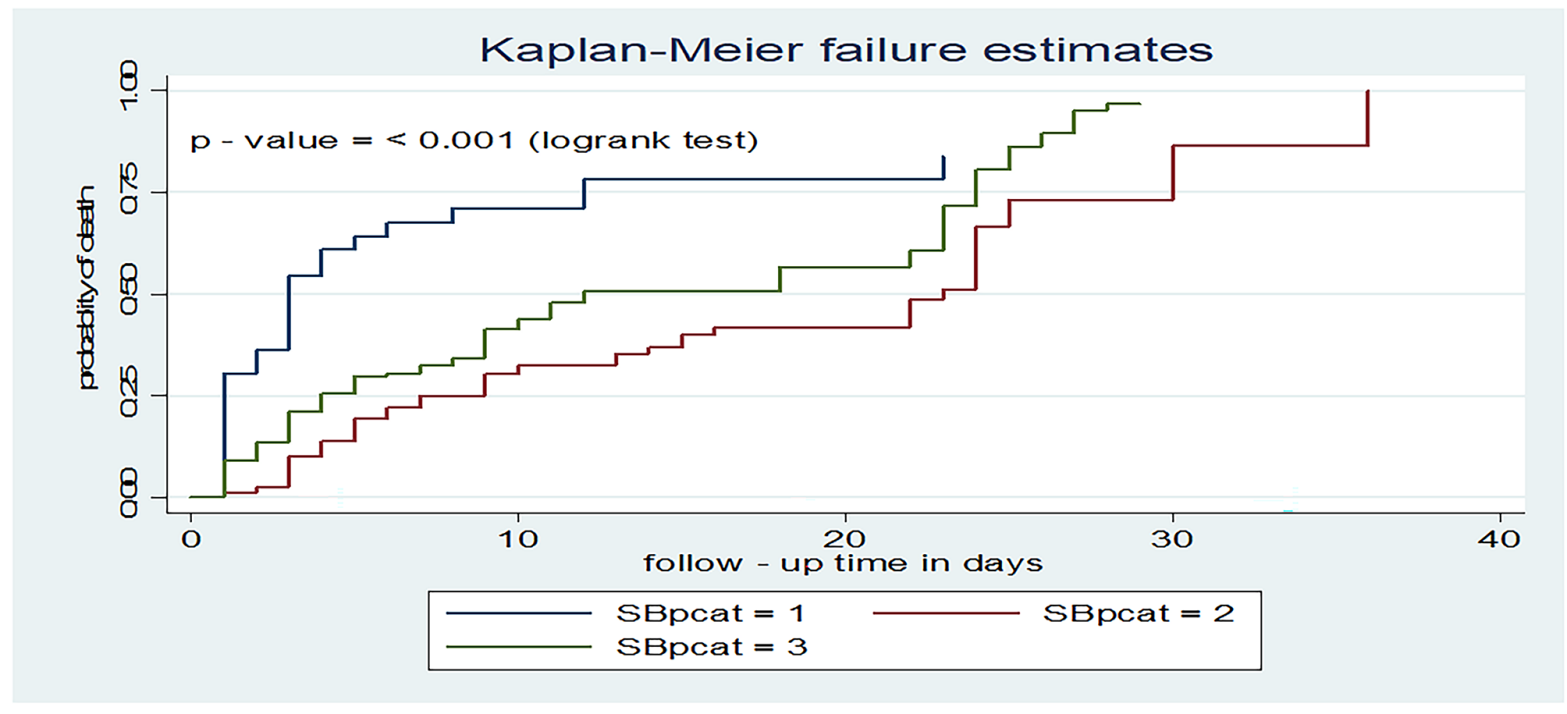
Kaplan-Meier failure curve for systolic blood pressure among adult patients on mechanical ventilation admitted to ICU comprehensive specialized hospitals in West Amhara

Moreover, those hypotensive patients at admission had a shorter time to death with a median survival time of 3 days (95% CI: 2, 6) as compared to normotensive patients (23 days, 95% CI: 15, 24). Similarly, patients having a higher systolic blood pressure (> 140mmHg) had also a shorter time to death with a median survival time of 12 days (95% CI: 10, 22) compared to normotensive patients (Fig. 7).

### Predictors of time to death among mechanically ventilated patients admitted to the ICU

Based on the multivariable Cox proportional hazard regression analysis, patients who underwent tracheostomy, CPR, SBP < 90 mmHg, and respiratory rate < 12 rate/min were significant predictors of time to death among mechanically ventilated patients admitted to the ICU.

Keeping other variables constant, underwent tracheostomy procedure among mechanically ventilated patients decreased the hazard of death by 60% as compared to their counterparts (AHR: 0.40, 95% CI: 0.20, 0.80). The hazard of death among those cardiopulmonary resuscitated patients was 8.8 times higher as compared to their counterparts (AHR: 8.78, 95% CI: 5.38, 14.35). Moreover, those mechanically ventilated patients who had hypotension at admission had 2.96 times a higher hazard of death as compared to those patients who were normotensive (AHR: 2.96, 95% CI: (1.11–7.87). Similarly, those patients who had a respiratory rate of < 12 breath/min had 2.7 times higher hazard of death as compared to those who had a respiratory rate of 12–20 breath/min (AHR: 2.74, 95% CI: (1.48–5.07)) (Table 8).

**Table 8:** Bivariable and multivariable Cox-proportional hazard regression analysis of predictors of time to death among mechanically ventilated patients admitted to the ICU in West Amhara, Comprehensive specialized hospitals, Ethiopia, 2022

Variables	Categories	Outcome		CHR (95%CI)	AHR (95% CI)
		Death	Censored		
Tracheostomy	Yes	18	15	0.57(0.40–0.82)	0.40(0.20–0.80)*
	No	225	133	1	1
CPR	Yes	161	14	4.51(3.41–5.9)	8.78(5.38–14.35)**
	No	82	134	1	1
SBP at admission (mmHg)	< 90	146	32	1.99(1.36–2.89)	2.96(1.11–7.87)*
	90–140	36	20	1	1
	> 140	61	96	0.67(0.48–0.95)	1.07(0.48–2.40)
RR at admission (rate/minute)	< 12	131	98	1.86(1.38–2.49)	2.74(1.48–5.07)*
	12–20	50	10	1	1
	> 20	62	40	1.44(0.74–2.80)	0.73(0.25- 2.11)

NB: * = *p*-value < 0.05, ** = *p*- value < 0.001; AHR = Adjusted Hazard Ratio, CHR = Crude Hazard Ratio, SBP = Systolic Blood pressure, RR = Respiratory rate, mmHg = Millimeter Mercury

## Discussion

This study aimed to determine the time to death and identify its predictors among patients on MV admitted to the ICU. According to the current study, the median time to death among patients on mechanical ventilator was 16 days. Those mechanically ventilated patients who underwent tracheostomy, received CPR, having a respiratory rate less than 12 breaths per minute, and were hypotensive were significant predictors for time to death.

This study found that the median time to death of mechanically ventilated ICU patients was 16 days (95% CI: 11–22). This finding is in line with the studies done in Saudi Arabia, Spain, and Ethiopia [5, 23, 24]. However, it is lower than the study conducted in Southern Brazil [2]. The reason for this discrepancy might be due to the difference in the quality of the health care delivery system [2, 25]. Additionally, it might also be due to the patients’ clinical status during admission to the ICU, which can increase the hazard of death [9, 25].

This study also showed that the overall incidence density rate of death among mechanically ventilated patients admitted to the ICU was 5.93 per 100-day observations (95% CI: 5.23–6.72), which is in line with the study conducted in Ethiopia [26]. The overall mortality of patients on mechanical ventilation was 62.2%, which is consistent with the previous study conducted in Ethiopia (60.7%) [27]. However, this finding is higher than the studies conducted in Ethiopia [24] Sub-Saharan Africa [28], and Brazil [2], which reported that the prevalence of mortality among mechanically ventilated patients admitted to the ICU was 33.78%, 49%, and 51%, respectively. The reason for this discrepancy might be due to the difference in the level of ICU organization and duration of follow-up.

Mechanically ventilated patients who underwent tracheostomy procedure had decreased the hazard of death by 60% as compared to their counterparts. This finding is supported by studies done in Nigeria and Taiwan [9, 29]. This might be due to the protective effects of tracheostomy procedure such as improved patient comfort, better oral hygiene, less dental damage and tracheal injury, easier and faster nursing care, and lower airway resistance, which may facilitate the weaning process and avoid ventilator-associated pneumonia [30].

This study also found that the hazard of death among those cardiopulmonary resuscitated patients was 8.8 times higher as compared to their counterparts. This finding is supported by the studies conducted in Ethiopia, Berlin, and France [24, 31, 32]. This might be due to the fact that those patients who were cardiopulmonary resuscitated had the problems of hemodynamic failure, lack of oxygenation [32], and a higher severity of illness [31], which facilitates patient mortality. The time of CPR initiation could be another possible reason since the chance of survival would be low if CPR couldn’t be initiated immediately after cardiac arrest due to severe and permanent brain damage [31, 33].

Mechanically ventilated patients who were hypotensive at admission had 2.96 times a higher hazard of death as compared to those who were normotensive. This is supported by studies done in France and China [32, 34]. This might be due to the scientific evidence that hypotension decreases tissue perfusion, which leads to multi-organ failure such as renal failure, cardiac failure, and brain death [35, 36]. Therefore, a healthcare provider should focus on maintaining the blood pressure of critically ill patients who have received mechanical ventilation.

Moreover, this study revealed that those patients who had a respiratory rate less than 12 breaths per minute had 2.7 times higher hazard of death as compared with those who had a respiratory rate of 12–20 breaths per minute. This is supported by the studies done in Denmark and Sweden [37, 38]. The possible reason for this might be attributed to the decreasing respiratory rate, which is an indicator of brain dysfunction from many causes, like structural or non-structural processes affecting the central nervous system, which leads to death [37, 38]. Therefore, measuring the respiratory rate is important for early identification of high-risk patients because the respiratory rate is a superior indicator to other physiologic parameters.

### Strengths and limitations of the study

This is a multi-center follow-up study, which increases its generalizability to the target population. However, this study has some limitations. Since it was institutional-based and the data were obtained from patients’ medical records, mortality-predicting variables such as the Acute Physiology and Chronic Health Evaluation (APACHE) and Sequential Organ Failure Assessment (SOFA) scores couldn’t be addressed.

## Conclusion

Patients treated with mechanical ventilation had a shorter time to death. Being cardiopulmonary resuscitated, hypotensive, and had lower respiratory rate were significant predictors of time to death, whereas patients who underwent tracheostomy was negatively associated with time to death. Therefore, we strongly recommended that the healthcare provider should perform a tracheostomy procedure early for critically ill patients who need prolonged ventilation, and a special attention should be given for patients who had lower systolic blood pressure and respiratory rate. Furthermore, a prospective follow up study is required by incorporating the Acute Physiology and Chronic Health Evaluation (APACHE) and Sequential Organ Failure Assessment (SOFA) scores to predict the mortality of mechanically ventilated patients.

### Electronic supplementary material

Below is the link to the electronic supplementary material.

Supplementary material 1: Data extraction checklist.

Supplementary material 2: Stata data set.

## Data Availability

Data will be available upon request from the corresponding author.

## Abbreviations

AHR: Adjusted Hazard Ratio
ARDS: Acute Respiratory Distress Syndrome
CHR: Crude Hazard Ratio
COPD: Chronic Obstructive Pulmonary Diseases
CI: Confidence Interval
GCS: Glasgow Coma Scale
IDR: Incidence Density Rate
KM: Kaplan Meier
LOS: Length Of Stay
MOF: Multi-Organ Failure
MV: Mechanical Ventilation
WHO: World Health Organization
