# Supplementary material for: Time to death and its predictors among adult patients on mechanical ventilation admitted to intensive care units in West Amhara comprehensive specialized hospitals, Ethiopia: a retrospective follow-up study
Source: BMC Anesthesiol. 2024 Mar 23;24:114. doi: 10.1186/s12871-024-02495-9 (PMC10960484; doi:10.1186/s12871-024-02495-9)
Supplement: Supplementary file 1 — Supplementary material 1: Data extraction checklist. [file 12871_2024_2495_MOESM1_ESM.docx]

# **ANNEX - 1:** **Data extraction tool**

| **Part I- Predictors present at the initiation of mechanical ventilation** | | |
| --- | --- | --- |
| S.No | Variable | Answer |
| 1.1 | Age | ___________years |
| 1.2 | Gender | 1. Male 2. Female |
| 1.3 | Residence | 1. Urban 2. Rural |
| **Part II- Clinical-related characteristics** | | |
| 1.4 | Diagnosis during admission to the ICU (disorder) | 1. Respiratory 2. Hematologic and Neuromuscular 3. Cardiovascular and Renal 4. Neurologic 5. Gastrointestinal 6. Other |
| 1.5 | Glasgow coma scale at admission | __________________ |
| 1.6 | Patient type | 1. Medical 2. Surgical 3. Obstetrics 4. Emergency |
| 1.7 | If the answer for no 1.6 is surgical, which one is from the following | 1. Emergent 2. Elective |
| 1.8 | Admission source to ICU | 1. Emergency 2. Ward 3. Operating room 4. Referred from other hospital |
| 1.9 | Has the patient comorbid illness | 1. Yes 2. No |
| 1.10 | If the answer for no 1.9, is yes which one  (disorder) | 1. Hepatic and renal 2. Cardiovascular and hematologic 3. Respiratory 4. Other |
| 1.11 | Does the patient experience the diagnosis of Acute respiratory distress syndrome | 1. Yes 2. No |
| 1.12 | Does the patient occurred with the diagnosis of pneumonia | 1. Yes 2. No |
| 1.13 | If the answer is yes for no 1.12, please write the type | ___________ |
| **Part III- Management-related characteristics** | | |
| 2.1 | Does the patient was using vasopressors | 1. Yes 2. No |
| 2.2 | Does the patient was using neuromuscular blockers during ICU stay | 1. Yes 2. No |
| 2.3 | Does the patient had put on tracheostomy procedure | 1. Yes 2. No |
| 2.4 | Does CPR performed | 1. Yes 2. No |
| 2.5 | Does dialysis procedure have been performed | 1. Yes 2. No |
| **Part IV- Mechanical ventilation-related predictors** | | |
| 3.1 | Cause of the initiation of MV | 1. Acute respiratory failure 2. Neurological condition (coma, shock stroke…) 3. Neuromuscular disease (tetanus, myasthenia gravis…) 4. Cardiovascular, renal and Hematologic |
| 3.2 | If the answer for number 3.1 is ARF, what causes it | 1. Post-operative complication 2. Road traffic accident 3. Pneumonia 4. Bronchial asthma 5. CHF 6. Cardiac arrest 7. Other |
| 3.3 | What was mode of mechanical ventilation at admission |  |
| 3.4 | Does the patient exposed to prolonged mechanical ventilation | 1. Yes 2. No |
| 3.5 | What was the weaning mode of mechanical ventilation |  |
| 3.6 | Does mechanical ventilator related complication occurred | 1. Yes 2. No |
| 3.7 | What was the outcome of the patient who received mechanical ventilation | 1. Death 2. Discharge 3. Transfer to the inpatient ward 4. Referred 5. Left against medical treatment |
| **Part V –Vital signs at admission** | | |
| 4.1 | Systolic Blood pressure | ___________ |
| 4.2 | Diastolic Blood pressure | __________ |
| 4.3 | Heart rate | __________ |
| 4.4 | Respiratory rate | ___________ |
| 4.5 | SPO2 | ___________ |
| 4.6 | Temperature in ^0^C |  |
