# Supplementary material for: Time to death and its predictors among adult patients on mechanical ventilation admitted to intensive care units in West Amhara comprehensive specialized hospitals, Ethiopia: a retrospective follow-up study
Source: BMC Anesthesiol. 2024 Mar 23;24:114. doi: 10.1186/s12871-024-02495-9 (PMC10960484; doi:10.1186/s12871-024-02495-9)
Supplement: Supplementary file 2 — Supplementary material 2: Stata data set. [file 12871_2024_2495_MOESM2_ESM.pdf]

ID	Age	Gender	Residence	Dx	GCS	ward	dmisssion sourc	vasopressor	previous mv
1	68	female	Urban	Neurological	15	medical	ward	no	no
2	68	female	Urban	Neurological	15	medical	ward	no	no
3	37	female	Urban	Respiratory	15	medical	emergency	no	no
4	18	male	Rural	Tetanus	15	medical	ward	no	yes
5	27	female	Urban	Respiratory	15	obstestrics	reffered	no	no
6	22	male	Rural	Neurological	4	medical	reffered	no	no
7	55	male	Rural	Neuromescular	5	Surgical	emergency	no	no
8	30	male	Rural	Gastro intestina	4	Surgical	operating room	no	no
9	58	male	Urban	Tetanus	7	medical	emergency	yes	yes
10	62	female	Rural	Tetanus	15	medical	emergency	yes	yes
11	48	male	Urban	Renal	15	Surgical	emergency	no	no
12	65	male	Rural	Tetanus	15	medical	ward	no	no
13	27	male	Rural	Renal	15	medical	ward	no	no
14	50	female	Rural	Neuromescular	9	medical	ward	yes	no
15	54	male	Urban	Neurological	3	medical	emergency	no	no
16	18	male	Rural	Neurological	3	medical	emergency	no	no
17	19	male	Rural	Respiratory	15	medical	emergency	no	no
18	42	male	Urban	Respiratory	6	medical	emergency	yes	no
19	58	female	Urban	Tetanus	15	medical	ward	no	no
20	18	female	Urban	Respiratory	10	medical	emergency	no	no
21	23	female	Rural	Neurological	7	medical	ward	no	no
22	40	male	Rural	Gastro intestina	15	Surgical	ward	no	no
23	26	male	Urban	Accident	3	Emergency	emergency	no	no
24	35	female	Rural	Respiratory	8	obstestrics	ward	no	no
25	32	female	Rural	Neurological	3	obstestrics	emergency	no	no
26	23	female	Rural	Neurological	3	medical	emergency	no	no
27	46	female	Rural	Tetanus	13	obstestrics	emergency	no	yes
28	60	female	Rural	Respiratory	15	medical	emergency	no	no
29	26	female	Urban	Tetanus	11	obstestrics	emergency	no	no
30	28	male	Rural	Accident	10	Surgical	ward	yes	yes
31	48	female	Urban	Respiratory	3	medical	ward	yes	no
32	40	male	Urban	Cardiovascular	7	medical	emergency	yes	no
33	30	female	Rural	Other	15	Surgical	ward	yes	yes
34	30	female	Rural	Cardiovascular	7	medical	emergency	yes	no
35	38	male	Urban	Tetanus	15	medical	emergency	yes	no
36	70	male	Rural	Respiratory	15	medical	reffered	yes	yes
37	33	male	Urban	Neurological	4	medical	reffered	no	no
38	70	male	Rural	Gastro intestina	9	Surgical	ward	no	no
39	18	male	Rural	Accident	7	Emergency	emergency	no	no
40	52	male	Rural	Respiratory	10	medical	reffered	no	no
41	22	male	Rural	Accident	3	Emergency	emergency	no	no
42	55	male	Rural	Respiratory	15	medical	emergency	no	no
43	27	male	Rural	Neurological	3	medical	emergency	no	no
44	23	male	Rural	Neurological	15	medical	ward	no	no
45	30	female	Urban	Respiratory	15	medical	emergency	no	no
46	61	male	Urban	Respiratory	15	medical	emergency	no	no

47	38	female	Urban	Respiratory	15	medical	ward	no	no
48	25	female	Rural	Respiratory	12	medical	reffered	no	no
49	32	female	Rural	Neurological	3	medical	emergency	yes	no
50	37	male	Rural	Neurological	3	medical	emergency	yes	no
51	52	female	Urban	Accident	15	medical	reffered	no	yes
52	20	male	Rural	Accident	5	Surgical	reffered	no	yes
53	30	male	Rural	Accident	4	medical	emergency	yes	no
54	18	male	Urban	Accident	8	medical	emergency	no	no
55	20	male	Rural	Respiratory	15	medical	emergency	no	no
56	19	male	Rural	Other	4	medical	reffered	no	no
57	52	female	Urban	Cardiovascular	13	medical	emergency	no	no
58	30	female	Rural	Respiratory	15	medical	reffered	no	no
59	26	male	Rural	Gastro intestina	15	Surgical	ward	no	no
60	30	female	Rural	Other	15	medical	reffered	no	no
61	92	male	Urban	Respiratory	15	medical	emergency	no	no
62	33	male	Urban	Respiratory	15	medical	ward	no	no
63	47	female	Rural	Respiratory	3	medical	emergency	no	no
64	23	female	Urban	Respiratory	6	medical	reffered	no	no
65	50	female	Urban	Gastro intestina	5	medical	reffered	no	no
66	35	female	Urban	Respiratory	12	medical	reffered	yes	no
67	25	male	Urban	Respiratory	3	medical	emergency	yes	no
68	18	female	Urban	Cardiovascular	5	medical	ward	yes	no
69	35	male	Urban	Accident	6	medical	reffered	no	no
70	18	male	Rural	Gastro intestina	3	medical	emergency	no	no
71	55	male	Rural	Respiratory	7	medical	ward	no	no
72	60	female	Urban	Cardiovascular	14	medical	ward	no	no
73	68	female	Rural	Cardiovascular	10	medical	emergency	yes	no
74	30	male	Rural	Respiratory	14	Surgical	operating room	no	no
75	68	female	Rural	Neurological	3	medical	emergency	no	yes
76	40	male	Rural	Gastro intestina	5	Surgical	emergency	no	no
77	54	male	Urban	Respiratory	15	medical	reffered	no	no
78	23	male	Rural	Other	3	medical	reffered	no	no
79	30	female	Rural	Hematologic	15	medical	ward	no	no
80	72	male	Urban	Respiratory	15	medical	reffered	yes	no
81	24	male	Urban	Respiratory	15	medical	ward	yes	no
82	29	female	Rural	Respiratory	5	obstestrics	reffered	yes	no
83	54	female	Rural	Neurological	5	medical	reffered	yes	yes
84	70	male	Rural	Respiratory	6	medical	reffered	yes	yes
85	37	male	Urban	Gastro intestina	10	Surgical	reffered	no	no
86	39	female	Urban	Respiratory	15	medical	emergency	no	no
87	51	female	Rural	Other	3	medical	reffered	no	no
88	55	male	Rural	Respiratory	15	medical	ward	no	no
89	66	male	Rural	Cardiovascular	6	Surgical	operating room	no	no
90	26	female	Rural	Cardiovascular	8	obstestrics	reffered	no	no
91	26	male	Rural	Neurological	3	medical	ward	no	no
92	24	female	Rural	Cardiovascular	14	medical	emergency	no	no
93	28	male	Urban	Accident	6	Emergency	emergency	no	no

94	35	female	Rural	Respiratory	14	obstestrics	ward	no	no
95	32	male	Rural	Accident	15	Surgical	ward	no	no
96	72	male	Rural	Respiratory	15	medical	ward	no	no
97	25	female	Rural	Neurological	4	obstestrics	reffered	no	no
98	42	male	Urban	Respiratory	15	medical	ward	no	no
99	35	female	Rural	Respiratory	15	medical	emergency	no	no
100	45	female	Urban	Cardiovascular	7	medical	emergency	no	no
101	60	female	Rural	Respiratory	12	medical	reffered	no	no
102	53	female	Rural	Respiratory	15	medical	emergency	no	no
103	25	male	Urban	Cardiovascular	15	medical	emergency	no	yes
104	49	female	Rural	Respiratory	5	medical	reffered	yes	no
105	45	male	Rural	Accident	7	medical	reffered	no	no
106	30	female	Rural	Accident	6	medical	reffered	no	no
107	64	male	Rural	Respiratory	3	medical	reffered	no	no
108	24	male	Rural	Neurological	3	medical	emergency	no	no
109	21	male	Urban	Tetanus	15	medical	emergency	yes	no
110	28	female	Rural	Respiratory	13	obstestrics	reffered	yes	no
111	26	male	Urban	Other	3	obstestrics	operating room	no	no
112	48	male	Rural	Accident	3	Surgical	reffered	yes	no
113	72	male	Urban	Respiratory	12	medical	reffered	no	no
114	59	male	Rural	Respiratory	11	medical	reffered	yes	no
115	23	male	Rural	Other	3	medical	ward	no	no
116	51	male	Rural	Gastro intestina	9	Surgical	ward	no	no
117	57	male	Rural	Neuromescular	15	medical	emergency	no	no
118	38	female	Urban	Respiratory	4	medical	ward	no	no
119	32	female	Rural	Respiratory	8	medical	reffered	no	no
120	21	male	Rural	Other	7	medical	ward	yes	no
121	30	male	Urban	Accident	5	Surgical	ward	no	yes
122	50	male	Rural	Gastro intestina	15	Surgical	operating room	no	no
123	27	female	Rural	Cardiovascular	14	obstestrics	reffered	no	no
124	24	male	Rural	Gastro intestina	15	Surgical	ward	no	no
125	55	female	Urban	Respiratory	15	medical	emergency	no	no
126	33	female	Urban	Other	11	obstestrics	ward	no	no
127	24	male	Urban	Gastro intestina	15	Surgical	reffered	no	no
128	32	female	Rural	Respiratory	15	obstestrics	ward	no	yes
129	40	male	Urban	Hematologic	15	medical	emergency	no	no
130	68	male	Rural	Neurological	6	medical	reffered	no	no
131	28	male	Urban	Neuromescular	15	medical	reffered	yes	no
132	60	male	Rural	Neurological	3	medical	reffered	yes	no
133	47	female	Rural	Other	15	obstestrics	reffered	no	no
134	50	female	Rural	Other	15	Surgical	ward	no	no
135	22	male	Urban	Accident	9	Emergency	emergency	no	no
136	41	male	Urban	Accident	5	Emergency	reffered	yes	no
137	70	female	Rural	Accident	6	Emergency	emergency	no	no
138	35	male	Rural	Cardiovascular	15	medical	emergency	no	no
139	55	female	Rural	Cardiovascular	15	medical	emergency	yes	no
140	70	female	Urban	Cardiovascular	15	medical	reffered	no	no

141	55	male	Urban	Respiratory	3	medical	emergency	yes	no
142	60	male	Urban	Accident	9	Emergency	ward	yes	yes
143	24	male	Rural	Gastro intestina	15	Surgical	operating room	yes	no
144	22	female	Rural	Other	15	obstestrics	ward	no	no
145	25	male	Rural	Cardiovascular	10	medical	emergency	yes	no
146	26	female	Urban	Hematologic	3	medical	emergency	yes	no
147	39	male	Urban	Accident	7	Surgical	reffered	no	yes
148	56	male	Rural	Neurological	5	medical	reffered	no	no
149	26	male	Urban	Respiratory	14	medical	emergency	no	no
150	29	male	Rural	Accident	5	Emergency	reffered	yes	no
151	66	male	Urban	Gastro intestina	10	Surgical	ward	no	yes
152	51	male	Urban	Accident	4	Emergency	emergency	no	no
153	33	female	Urban	Respiratory	15	medical	reffered	no	no
154	40	male	Rural	Neurological	10	Surgical	ward	no	yes
155	45	female	Rural	Cardiovascular	15	medical	reffered	yes	no
156	54	male	Urban	Neuromescular	15	medical	reffered	yes	yes
157	24	male	Urban	Accident	6	Emergency	reffered	no	no
158	64	male	Rural	Accident	15	medical	emergency	yes	no
159	22	female	Rural	Respiratory	15	medical	emergency	no	no
160	27	female	Rural	Renal	15	medical	reffered	no	no
161	19	female	Rural	Cardiovascular	15	obstestrics	reffered	yes	no
162	41	male	Rural	Neuromescular	15	Surgical	ward	no	no
163	62	male	Rural	Gastro intestina	15	Surgical	ward	yes	no
164	75	male	Urban	Respiratory	15	medical	reffered	no	no
165	25	male	Urban	Neuromescular	15	medical	emergency	no	no
166	29	male	Urban	Other	10	medical	emergency	yes	no
167	24	female	Urban	Accident	11	medical	reffered	no	no
168	42	male	Urban	Neurological	6	medical	reffered	no	no
169	65	male	Rural	Respiratory	15	medical	reffered	no	no
170	47	female	Rural	Accident	8	Surgical	ward	no	no
171	66	male	Urban	Renal	15	medical	reffered	yes	no
172	44	male	Urban	Cardiovascular	12	medical	ward	yes	no
173	30	female	Rural	Other	3	medical	reffered	yes	no
174	32	male	Urban	Respiratory	7	medical	reffered	yes	no
175	80	female	Urban	Neuromescular	15	Surgical	reffered	no	no
176	65	female	Urban	Respiratory	15	medical	emergency	no	no
177	30	male	Rural	Accident	6	Surgical	operating room	no	yes
178	19	male	Urban	Respiratory	15	Surgical	emergency	no	no
179	42	female	Rural	Accident	10	Surgical	emergency	no	yes
180	75	female	Urban	Accident	8	Surgical	reffered	no	no
181	29	female	Urban	Respiratory	15	medical	emergency	no	no
182	64	male	Rural	Neurological	10	medical	ward	no	no
183	60	female	Urban	Neurological	9	medical	emergency	no	no
184	26	female	Rural	Neurological	3	medical	emergency	yes	no
185	36	male	Rural	Neurological	3	medical	emergency	yes	no
186	35	male	Rural	Accident	3	medical	emergency	no	no
187	34	female	Urban	Neurological	4	obstestrics	emergency	no	no

188	62	male	Urban	Respiratory	13	medical	ward	yes	no
189	61	male	Urban	Respiratory	15	medical	emergency	no	no
190	41	female	Rural	Neurological	5	medical	reffered	yes	no
191	48	male	Rural	Tetanus	15	medical	ward	no	no
192	28	male	Urban	Gastro intestina	15	Surgical	operating room	no	yes
193	36	male	Urban	Accident	6	Emergency	emergency	no	no
194	56	male	Urban	Gastro intestina	15	Surgical	ward	no	no
195	31	female	Rural	Neurological	9	medical	ward	no	no
196	46	female	Rural	Accident	11	Emergency	emergency	no	no
197	23	female	Rural	Other	15	medical	reffered	yes	no
198	41	male	Urban	Cardiovascular	5	medical	reffered	yes	no
199	84	male	Urban	Respiratory	6	Surgical	emergency	no	no
200	63	female	Rural	Hematologic	7	medical	ward	yes	yes
201	82	male	Urban	Neuromescular	4	medical	emergency	no	yes
202	48	female	Rural	Respiratory	15	medical	reffered	no	no
203	37	male	Urban	Cardiovascular	15	medical	ward	no	no
204	58	female	Rural	Neurological	3	medical	emergency	yes	yes
205	74	male	Urban	Renal	13	medical	reffered	no	no
206	25	female	Rural	Accident	6	Emergency	emergency	no	no
207	56	male	Urban	Gastro intestina	14	Surgical	emergency	no	yes
208	60	male	Rural	Renal	15	medical	ward	yes	yes
209	78	male	Urban	Respiratory	15	medical	emergency	yes	yes
210	71	male	Urban	Respiratory	15	medical	emergency	no	no
211	86	female	Rural	Other	5	medical	emergency	yes	no
212	38	male	Urban	Hematologic	12	medical	ward	no	no
213	81	male	Rural	Respiratory	8	medical	reffered	yes	yes
214	55	male	Urban	Neuromescular	15	medical	emergency	yes	yes
215	78	female	Rural	Neurological	3	medical	reffered	no	yes
216	67	male	Urban	Renal	6	medical	ward	yes	yes
217	47	male	Rural	Respiratory	9	obstestrics	operating room	no	yes
218	86	male	Rural	Cardiovascular	15	medical	operating room	no	no
219	38	female	Urban	Gastro intestina	15	Surgical	emergency	no	yes
220	53	male	Urban	Accident	4	Emergency	emergency	yes	yes
221	66	male	Rural	Tetanus	15	medical	ward	no	yes
222	28	male	Urban	Other	5	medical	reffered	no	no
223	74	male	Rural	Neurological	3	medical	reffered	yes	yes
224	90	male	Urban	Cardiovascular	4	medical	emergency	no	no
225	68	female	Rural	Cardiovascular	6	Surgical	operating room	no	no
226	70	male	Urban	Respiratory	5	Surgical	operating room	no	yes
227	83	male	Urban	Respiratory	5	medical	operating room	no	yes
228	50	male	Urban	Accident	5	Emergency	emergency	no	no
229	48	male	Rural	Tetanus	15	medical	ward	no	yes
230	62	female	Rural	Neurological	7	medical	reffered	no	yes
231	56	male	Rural	Tetanus	12	medical	ward	no	yes
232	35	male	Rural	Other	5	medical	reffered	no	no
233	78	male	Urban	Cardiovascular	15	medical	emergency	no	no
234	39	male	Urban	Other	11	medical	reffered	no	yes

235	74	male	Urban	Cardiovascular	3	medical	emergency	yes	yes
236	86	male	Urban	Neurological	4	medical	emergency	no	yes
237	69	female	Rural	Cardiovascular	3	medical	reffered	no	yes
238	54	male	Rural	Tetanus	8	medical	ward	no	yes
239	68	male	Urban	Respiratory	6	medical	emergency	no	yes
240	78	male	Urban	Neurological	4	medical	reffered	no	yes
241	83	female	Rural	Cardiovascular	5	medical	emergency	no	no
242	23	female	Rural	Other	15	medical	reffered	yes	no
243	41	male	Urban	Cardiovascular	5	medical	reffered	yes	no
244	84	male	Urban	Respiratory	6	Surgical	emergency	no	no
245	63	female	Rural	Hematologic	7	medical	ward	yes	yes
246	82	male	Urban	Neuromescular	4	medical	emergency	no	yes
247	48	female	Rural	Respiratory	15	medical	reffered	no	no
248	37	male	Urban	Cardiovascular	15	medical	ward	no	no
249	58	female	Rural	Neurological	3	medical	emergency	yes	yes
250	74	male	Urban	Renal	13	medical	reffered	no	no
251	25	female	Rural	Accident	6	Emergency	emergency	no	no
252	56	male	Urban	Gastro intestina	14	Surgical	emergency	no	yes
253	60	male	Rural	Renal	15	medical	ward	yes	yes
254	78	male	Urban	Respiratory	15	medical	emergency	yes	yes
255	71	male	Urban	Respiratory	15	medical	emergency	no	no
256	86	female	Rural	Other	5	medical	emergency	yes	no
257	38	male	Urban	Hematologic	12	medical	ward	no	no
258	81	male	Rural	Respiratory	8	medical	reffered	yes	yes
259	55	male	Urban	Neuromescular	15	medical	emergency	yes	yes
260	78	female	Rural	Neurological	3	medical	reffered	no	yes
261	67	male	Urban	Renal	6	medical	ward	yes	yes
262	47	male	Rural	Respiratory	9	obstestrics	operating room	no	yes
263	86	male	Rural	Cardiovascular	15	medical	operating room	no	no
264	38	female	Urban	Gastro intestina	15	Surgical	emergency	no	yes
265	53	male	Urban	Accident	4	Emergency	emergency	yes	yes
266	66	male	Rural	Tetanus	15	medical	ward	no	yes
267	28	male	Urban	Other	5	medical	reffered	no	no
268	74	male	Rural	Neurological	3	Surgical	reffered	yes	yes
269	90	male	Urban	Cardiovascular	4	medical	emergency	no	no
270	68	female	Rural	Cardiovascular	6	Surgical	operating room	no	no
271	70	male	Urban	Respiratory	5	Surgical	operating room	no	yes
272	83	male	Urban	Respiratory	5	medical	operating room	no	yes
273	50	male	Urban	Accident	5	Emergency	emergency	no	no
274	48	male	Rural	Tetanus	15	medical	ward	no	yes
275	62	female	Rural	Neurological	7	medical	reffered	no	yes
276	56	male	Rural	Tetanus	12	medical	ward	no	yes
277	35	male	Rural	Other	5	medical	reffered	no	no
278	78	male	Urban	Cardiovascular	15	medical	emergency	no	no
279	39	male	Urban	Other	11	medical	reffered	no	yes
280	74	male	Urban	Cardiovascular	3	medical	emergency	yes	yes
281	86	male	Urban	Neurological	4	medical	emergency	no	yes

282	69	female	Rural	Cardiovascular	3	medical	reffered	no	yes
283	54	male	Rural	Tetanus	8	medical	ward	no	yes
284	68	male	Urban	Neurological	11	medical	ward	no	yes
285	73	male	Urban	Neurological	4	medical	reffered	no	yes
286	83	female	Rural	Cardiovascular	5	medical	emergency	no	no
287	68	female	Urban	Neurological	15	medical	ward	no	no
288	18	male	Rural	Tetanus	15	medical	ward	no	yes
289	22	male	Rural	Neurological	4	medical	reffered	no	no
290	55	male	Rural	Neuromescular	5	Surgical	ward	no	no
291	30	male	Rural	Gastro intestina	4	Surgical	operating room	no	no
292	58	male	Urban	Tetanus	7	medical	emergency	yes	yes
293	62	female	Rural	Tetanus	15	medical	emergency	yes	yes
294	48	male	Urban	Renal	15	Surgical	emergency	no	no
295	65	male	Rural	Tetanus	15	medical	ward	no	no
296	50	female	Rural	Neuromescular	9	medical	ward	yes	no
297	54	male	Urban	Cardiovascular	15	medical	ward	no	no
298	18	male	Rural	Neurological	3	medical	emergency	no	no
299	42	male	Urban	Respiratory	6	medical	emergency	yes	no
300	58	female	Urban	Tetanus	15	medical	ward	no	no
301	23	female	Rural	Neurological	7	medical	ward	no	no
302	40	male	Rural	Gastro intestina	15	Surgical	ward	no	no
303	56	male	Urban	Accident	3	Emergency	emergency	no	no
304	45	female	Rural	Respiratory	8	obstestrics	ward	no	no
305	42	female	Rural	Neurological	3	obstestrics	emergency	no	no
306	53	female	Rural	Neurological	3	medical	emergency	no	no
307	46	female	Rural	Tetanus	13	obstestrics	emergency	no	yes
308	60	female	Rural	Respiratory	15	medical	emergency	no	no
309	26	female	Urban	Tetanus	11	obstestrics	emergency	no	no
310	48	female	Urban	Respiratory	13	medical	ward	yes	no
311	50	male	Urban	Cardiovascular	7	medical	emergency	yes	no
312	30	female	Rural	Other	15	Surgical	ward	yes	yes
313	52	female	Rural	Cardiovascular	7	medical	emergency	yes	no
314	70	male	Rural	Respiratory	15	medical	reffered	yes	yes
315	53	male	Urban	Neurological	4	medical	reffered	no	no
316	70	male	Rural	Gastro intestina	9	Surgical	ward	no	no
317	68	male	Rural	Accident	7	Emergency	emergency	no	no
318	52	male	Rural	Respiratory	10	medical	reffered	no	no
319	55	male	Rural	Respiratory	15	medical	emergency	no	no
320	57	male	Rural	Neurological	3	medical	emergency	no	no
321	43	male	Rural	Neurological	15	medical	ward	no	no
322	61	male	Urban	Respiratory	15	medical	emergency	no	no
323	58	female	Urban	Respiratory	15	medical	ward	no	no
324	45	male	Rural	Respiratory	8	medical	reffered	no	no
325	52	female	Rural	Neurological	3	medical	emergency	yes	no
326	57	male	Rural	Neurological	3	medical	emergency	yes	no
327	52	female	Urban	Accident	5	medical	reffered	no	yes
328	51	male	Rural	Accident	5	Surgical	reffered	no	yes

329	43	male	Rural	Accident	1	medical	emergency	yes	no
330	50	male	Rural	Respiratory	15	medical	emergency	no	no
331	29	male	Rural	Other	4	medical	reffered	no	no
332	52	male	Urban	Cardiovascular	13	medical	emergency	no	no
333	92	male	Urban	Respiratory	15	medical	emergency	no	no
334	47	female	Rural	Respiratory	3	medical	emergency	no	no
335	50	female	Urban	Gastro intestina	5	medical	reffered	no	no
336	45	female	Urban	Respiratory	12	medical	reffered	yes	no
337	27	male	Urban	Respiratory	3	medical	emergency	yes	no
338	35	male	Urban	Accident	6	medical	reffered	no	no
339	55	male	Rural	Respiratory	7	medical	ward	no	no
340	60	female	Urban	Cardiovascular	14	medical	ward	no	no
341	68	female	Rural	Cardiovascular	10	medical	emergency	yes	no
342	68	female	Rural	Neurological	3	medical	emergency	no	yes
343	64	male	Urban	Respiratory	15	medical	reffered	no	no
344	23	male	Rural	Other	3	medical	reffered	no	no
345	72	male	Urban	Respiratory	15	medical	reffered	yes	no
346	64	male	Urban	Respiratory	15	medical	ward	yes	no
347	59	female	Rural	Respiratory	5	obstestrics	reffered	yes	no
348	62	male	Urban	Neurological	5	medical	reffered	yes	yes
349	70	male	Rural	Respiratory	6	medical	reffered	yes	yes
350	69	male	Urban	Respiratory	15	medical	emergency	no	no
351	61	female	Rural	Other	3	medical	reffered	no	no
352	65	male	Rural	Respiratory	15	medical	ward	no	no
353	66	male	Rural	Respiratory	6	Surgical	operating room	no	no
354	56	male	Rural	Neurological	3	medical	ward	no	no
355	72	male	Rural	Respiratory	15	medical	ward	no	no
356	25	female	Rural	Neurological	4	obstestrics	reffered	no	no
357	62	male	Urban	Respiratory	15	medical	ward	no	no
358	55	female	Urban	Cardiovascular	7	medical	emergency	no	no
359	60	female	Rural	Respiratory	12	medical	reffered	no	no
360	63	male	Urban	Respiratory	5	medical	emergency	no	no
361	60	female	Rural	Accident	6	medical	reffered	no	no
362	64	male	Rural	Respiratory	3	medical	reffered	no	no
363	24	male	Rural	Neurological	3	medical	emergency	no	no
364	72	male	Urban	Respiratory	12	medical	reffered	no	no
365	59	male	Rural	Respiratory	11	medical	reffered	yes	no
366	58	female	Urban	Respiratory	4	medical	ward	no	no
367	30	male	Urban	Accident	5	Surgical	ward	no	yes
368	68	male	Rural	Neurological	6	medical	reffered	no	no
369	60	male	Rural	Neurological	3	medical	reffered	yes	no
370	60	female	Rural	Other	15	Surgical	ward	no	no
371	70	female	Rural	Accident	6	Emergency	emergency	no	no
372	60	male	Urban	Accident	9	Emergency	ward	yes	yes
373	49	male	Urban	Accident	7	Surgical	reffered	no	yes
374	66	male	Rural	Neurological	5	medical	reffered	no	no
375	66	male	Urban	Gastro intestina	10	Surgical	ward	no	no

376	51	male	Urban	Accident	4	Emergency	emergency	no	no
377	54	male	Urban	Neuromescular	15	medical	reffered	yes	yes
378	24	male	Urban	Accident	6	Emergency	reffered	no	no
379	75	male	Urban	Respiratory	15	medical	reffered	no	no
380	65	male	Rural	Respiratory	15	medical	reffered	no	no
381	66	male	Urban	Renal	15	medical	reffered	yes	no
382	44	male	Urban	Cardiovascular	12	medical	ward	yes	no
383	80	female	Urban	Neuromescular	15	Surgical	reffered	no	no
384	65	female	Urban	Respiratory	15	medical	emergency	no	no
385	30	male	Rural	Accident	6	Surgical	operating room	no	yes
386	42	female	Rural	Accident	10	Surgical	emergency	yes	yes
387	75	female	Urban	Accident	8	Surgical	reffered	no	no
388	64	male	Urban	Neurological	9	medical	emergency	no	no
389	66	female	Rural	Neurological	3	medical	emergency	yes	no
390	62	male	Urban	Respiratory	3	medical	ward	yes	no
391	68	male	Urban	Gastro intestina	15	Surgical	operating room	no	yes

NB	comorbidity	Ifyes	OF	tracheostomy	metracheostom	ARDS	pnumonia	gth of hodpital	CU length of sta
yes	no		no	no		yes	no	14	8
yes	no		no	no		yes	no	14	8
no	yes	respiratory	no	no		no	no	10	10
no	no		no	yes	after intubation	no	no	39	29
no	yes	other	no	no		yes	no	4	4
no	no		no	no		no	no	13	13
no	no		yes	no		no	no	10	9
no	no		no	no		no	no	3	3
no	yes	renal	no	yes	after intubation	no	no	32	27
no	yes	renal	yes	yes	after intubation	no	no	36	28
no	yes	respiratory	yes	no		yes	no	3	3
no	no		no	no		no	yes	14	10
no	yes	renal	yes	no		no	yes	7	5
no	yes	other	no	no		no	yes	13	9
no	no		no	no		no	no	9	9
no	no		no	no		no	no	9	1
no	yes	respiratory	no	no		no	yes	6	6
no	yes	cardiovascular	yes	no		yes	yes	4	4
no	yes	other	no	no		no	yes	9	6
no	no		no	no		yes	yes	5	5
no	no		no	no		no	no	5	3
no	no		no	no		no	no	46	13
no	no		no	no		no	no	3	3
no	no		no	no		yes	yes	35	20
no	yes	cardiovascular	yes	no		no	no	3	3
no	yes	other	no	no		no	no	22	22
no	yes	other	no	no		no	yes	29	29
no	no		no	no		yes	yes	16	16
no	yes	hematologic	no	no		no	yes	13	11
yes	no		no	yes	after intubation	no	no	57	28
no	yes	other	yes	no		yes	no	27	11
no	yes	other	no	no		no	no	4	4
yes	yes	other	no	yes	after intubation	no	no	29	29
no	yes	cardiovascular	yes	no		no	no	1	1
no	no		no	yes	after intubation	no	no	4	4
no	no		yes	no		yes	yes	26	22
yes	yes	other	no	no		no	no	1	1
no	no		no	no		no	no	20	5
no	no		no	no		no	yes	1	1
no	no		no	no		yes	yes	1	1
no	no		no	no		no	yes	1	1
no	yes	respiratory	no	no		no	yes	4	4
no	no		no	no		no	no	6	6
no	yes	hepatic	no	no		no	no	15	5
no	yes	respiratory	no	no		no	no	3	3
no	no	hepatic	no	no		no	yes	1	1

no	yes	other	no	no		yes	yes	16	13
yes	no		no	no		yes	yes	18	18
no	no		no	no		yes	yes	1	1
no	yes	other	no	no		yes	yes	3	3
no	yes	other	no	yes	after intubation	no	yes	30	30
no	no		no	yes	after intubation	no	no	70	37
no	no		no	no		no	yes	3	3
no	no		no	no		no	yes	4	4
no	no		no	no		yes	yes	11	11
no	no		no	no		yes	yes	10	10
no	yes	cardiovascular	no	no		no	yes	6	6
no	yes	other	no	no		no	yes	12	12
no	no		no	no		no	yes	12	12
no	yes	respiratory	no	no		no	no	7	7
no	yes	other	no	no		no	yes	12	12
yes	no		no	yes	efore intubatio	no	no	8	7
no	no		no	no		no	no	9	9
no	no		no	no		no	no	2	2
yes	no		no	yes	after intubation	no	no	24	24
yes	yes	cardiovascular	no	no		yes	yes	20	20
yes	yes	hepatic	no	no		yes	yes	10	10
yes	yes	cardiovascular	no	no		no	yes	6	3
yes	no		yes	no		no	no	18	18
yes	no		no	no		no	no	13	13
yes	no		no	yes	after intubation	no	no	27	12
yes	yes	cardiovascular	yes	no		no	yes	28	5
yes	yes	cardiovascular	no	no		no	no	4	4
yes	no		no	yes	after intubation	no	no	3	2
yes	no		yes	no		no	no	34	34
yes	no		no	no		no	no	6	6
no	yes	other	no	no		yes	yes	15	15
no	yes	other	no	no		no	no	2	2
no	no		yes	no		yes	yes	11	9
no	no		no	no		yes	yes	2	2
no	no		yes	no		yes	yes	5	3
yes	no		no	no		yes	yes	3	3
no	yes	hematologic	no	yes	after intubation	no	no	28	28
no	no		no	no		no	no	37	37
no	no		no	no		no	no	1	1
no	yes	cardiovascular	no	no		yes	yes	1	1
no	no		yes	no		no	no	1	1
no	no		no	no		yes	yes	29	9
no	yes	other	no	no		no	no	9	5
no	no		no	no		no	no	4	4
no	no		no	no		no	yes	9	9
no	no		no	no		no	yes	5	5
no	no		no	no		no	yes	3	3

no	yes	other	no	no		no	yes	15	2
no	no		no	no		yes	no	11	2
no	no		yes	no		yes	yes	16	8
no	no		no	no		no	no	3	3
yes	yes	other	yes	no		no	yes	13	9
no	yes	respiratory	no	no		no	yes	6	6
no	yes	respiratory	no	no		no	yes	2	2
no	no		no	no		yes	yes	10	10
no	no		yes	no		no	yes	18	18
yes	no		no	no		yes	yes	1	1
no	no		yes	no		yes	no	3	3
yes	no		yes	no		no	yes	3	3
no	yes	other	no	no		no	no	15	15
no	no		no	no		yes	yes	1	1
no	yes	respiratory	no	no		no	yes	10	10
no	no		no	yes	efore intubatio	no	yes	10	10
no	no		no	no		yes	no	6	6
no	yes	hematologic	no	no		no	yes	7	7
no	no		no	no		no	yes	5	5
no	yes	cardiovascular	no	no		yes	yes	4	4
no	yes	renal	no	no		yes	yes	7	7
no	no		no	no		no	yes	6	5
no	no		no	no		no	no	9	7
no	yes	other	no	no		no	no	18	18
no	yes	other	yes	no		yes	yes	3	2
no	no		no	no		yes	yes	15	15
no	no		no	no		no	no	6	5
no	no		no	no		no	no	31	25
no	no		no	no		no	no	3	3
no	yes	other	yes	no		no	yes	4	4
no	no		no	no		no	no	15	23
no	no		no	no		yes	yes	3	3
no	no		yes	no		no	no	5	2
no	no		no	no		no	no	21	21
no	no	other	no	yes	after intubation	no	yes	26	24
yes	yes	other	no	yes	after intubation	no	no	27	18
no	no		no	no		no	no	5	5
no	yes	cardiovascular	yes	no		no	yes	8	8
no	yes	other	no	no		no	yes	5	5
no	yes	cardiovascular	no	no		no	yes	10	10
no	yes	other	no	no		no	no	4	1
no	no		no	no		no	no	9	9
no	no		no	no		no	no	2	2
no	no		no	no		no	no	14	14
no	yes	cardiovascular	no	no		no	no	8	8
no	no		no	no		yes	no	1	1
no	yes	cardiovascular	no	no		no	no	15	13

no	yes	renal	no	no		yes	yes	13	13
no	yes	other	no	no		no	yes	49	44
no	no		no	no		no	no	3	3
no	yes	other	no	no		no	yes	6	6
no	yes	cardiovascular	no	no		yes	yes	2	2
no	yes	renal	no	no		yes	yes	13	13
no	yes	cardiovascular	no	no		no	yes	28	23
no	yes	other	no	no		no	no	3	3
no	yes	hepatic	yes	no		no	yes	1	1
yes	no		no	no		no	no	2	2
no	no		no	no		no	no	14	3
no	no		no	no		no	yes	5	5
no	yes	respiratory	no	no		yes	yes	2	2
yes	no		no	no		no	no	42	34
no	yes	cardiovascular	yes	no		no	no	12	12
no	yes	other	yes	no		no	no	29	26
no	no		no	no		no	yes	10	10
no	no		no	no		no	no	6	6
no	yes	other	no	no		yes	yes	3	3
no	yes	cardiovascular	yes	no		no	no	14	10
yes	no		yes	no		no	no	1	1
no	no		no	no		no	no	11	5
yes	no		yes	no		no	no	1	1
no	no		no	no		yes	yes	4	4
no	yes	other	no	no		no	no	3	3
yes	no		no	no		no	yes	20	20
no	yes	cardiovascular	no	no		no	no	1	1
no	yes	other	yes	no		no	no	12	3
no	yes	renal	yes	no		no	yes	5	5
no	no		no	no		no	yes	11	5
no	yes	cardiovascular	yes	no		no	yes	8	8
no	no		yes	no		yes	yes	24	18
no	yes	other	no	no		no	no	9	9
no	yes	other	no	no		yes	yes	1	1
no	no		no	no		no	no	2	2
no	yes	other	yes	no		yes	yes	3	3
no	no		no	yes	after intubation	no	no	41	41
no	no		yes	yes	after intubation	no	no	26	26
no	no		no	yes	after intubation	no	no	27	27
no	yes	cardiovascular	no	no		no	no	28	10
no	yes	respiratory	no	no		yes	yes	20	18
no	yes	other	yes	no		no	yes	17	11
no	yes	cardiovascular	yes	no		no	no	1	1
no	yes	hepatic	no	no		no	yes	1	1
no	yes	other	yes	no		no	no	2	2
no	no		yes	no		no	yes	1	1
no	yes	other	yes	no		no	yes	1	1

no	yes	other	yes	no		yes	yes	19	9
no	yes	renal	no	no		yes	yes	2	2
no	yes	other	yes	no		no	yes	6	6
yes	no		yes	no		no	no	8	6
no	no		no	no		no	no	29	23
no	no		no	no		no	no	16	7
no	yes	other	yes	no		no	no	11	9
yes	yes	cardiovascular	no	no		no	no	27	19
no	no		no	no		no	yes	5	5
no	no		yes	no		no	no	1	1
no	no		yes	no		yes	no	1	1
no	yes	other	no	no		no	no	5	5
no	yes	respiratory	no	no		no	no	24	24
no	yes	hepatic	no	no		no	no	27	27
no	yes	respiratory	no	no		yes	yes	7	7
no	yes	renal	no	no		no	yes	12	12
no	yes	cardiovascular	yes	yes	after intubation	no	no	29	29
no	yes	cardiovascular	no	no		yes	yes	18	18
no	no		yes	no		no	no	7	7
no	no		no	no		no	no	25	25
no	yes	cardiovascular	yes	yes	after intubation	no	no	30	26
yes	yes	respiratory	no	no		yes	yes	25	25
no	yes	hematologic	no	no		yes	yes	17	17
yes	yes	other	yes	no		no	no	9	9
no	yes	other	no	no		no	no	5	5
no	yes	respiratory	no	yes	after intubation	no	no	33	33
no	yes	other	no	no		no	no	31	31
no	yes	other	no	no		no	no	26	26
no	yes	respiratory	no	yes	after intubation	no	no	28	28
no	yes	other	no	yes	after intubation	yes	yes	24	24
no	no		yes	no		no	no	35	29
no	yes	other	no	no		no	no	41	35
yes	yes	other	yes	yes	after intubation	no	no	31	31
yes	yes	other	no	yes	after intubation	no	no	32	29
yes	yes	other	no	no		no	yes	15	15
no	yes	hepatic	yes	no		no	no	23	23
no	yes	cardiovascular	no	no		no	no	18	18
no	yes	hepatic	no	no		no	no	9	9
no	yes	other	no	no		no	no	25	25
no	yes	respiratory	yes	no		yes	yes	43	32
no	yes	other	no	no		no	no	3	3
no	yes	other	no	yes	after intubation	no	no	30	30
no	yes	other	no	yes	after intubation	no	no	29	29
no	yes	other	no	no		no	no	26	23
no	no		no	no		no	yes	5	5
no	yes	cardiovascular	yes	no		yes	yes	8	8
no	yes	other	no	no		yes	yes	24	24

no	yes	other	no	no		no	no	22	22
no	yes	respiratory	no	no		no	no	25	25
no	yes	other	yes	no		yes	yes	27	27
no	yes	cardiovascular	no	yes	after intubation	no	no	36	32
no	yes	renal	yes	yes	after intubation	no	no	25	25
no	yes	cardiovascular	no	no		no	no	28	28
no	yes	other	yes	no		no	no	4	4
no	no		yes	no		no	no	1	1
no	no		yes	no		yes	no	1	1
no	yes	other	no	no		no	no	5	5
no	yes	respiratory	no	no		no	no	24	24
no	yes	hepatic	no	no		no	no	27	27
no	yes	respiratory	no	no		yes	yes	7	7
no	yes	cardiovascular	no	no		no	yes	12	12
no	yes	cardiovascular	yes	yes	after intubation	no	no	29	29
no	yes	cardiovascular	no	no		yes	yes	18	18
no	no		yes	no		no	no	7	7
yes	yes	renal	no	no		no	no	25	25
no	yes	cardiovascular	yes	yes	after intubation	no	no	30	26
yes	yes	respiratory	no	no		yes	yes	25	25
no	yes	hematologic	no	no		yes	yes	17	17
yes	yes	other	yes	no		no	no	9	9
no	yes	other	no	no		no	no	5	5
no	yes	respiratory	no	yes	after intubation	no	no	33	33
no	yes	other	no	no		no	no	31	31
no	yes	other	no	no		no	no	26	26
no	yes	respiratory	no	yes	after intubation	no	no	28	28
no	yes	other	no	yes	after intubation	yes	yes	24	24
no	no		yes	no		no	no	35	29
no	yes	other	no	no		no	no	41	35
yes	yes	other	yes	yes	after intubation	no	no	31	31
yes	yes	other	no	yes	after intubation	no	no	32	29
yes	yes	other	no	no		no	yes	15	15
no	yes	hepatic	yes	no		no	no	23	23
no	yes	cardiovascular	no	no		no	no	18	18
no	yes	hepatic	no	no		no	no	9	9
no	yes	other	no	no		no	no	25	25
no	yes	respiratory	yes	no		yes	yes	43	32
no	yes	other	no	no		no	no	3	3
no	yes	other	no	yes	after intubation	no	no	30	30
no	yes	other	no	yes	after intubation	no	no	29	29
no	yes	other	no	no		no	no	26	23
no	no		no	no		no	yes	5	5
no	yes	cardiovascular	yes	no		yes	yes	8	8
no	yes	other	no	no		yes	yes	24	24
no	yes	other	no	no		no	no	22	22
no	yes	respiratory	no	no		no	no	25	25

no	yes	other	yes	no		yes	yes	27	27
no	yes	cardiovascular	no	yes	after intubation	no	no	36	32
no	yes	respiratory	yes	yes	after intubation	no	no	25	25
no	yes	cardiovascular	no	no		no	no	28	28
no	yes	other	yes	no		no	no	4	4
yes	no		no	no		yes	no	14	8
no	no		no	yes	after intubation	no	no	39	29
no	no		no	no		no	no	13	13
no	no		yes	no		no	no	10	9
no	no		no	no		no	no	3	3
no	yes	renal	yes	no		no	no	32	27
no	yes	renal	yes	yes	after intubation	no	no	36	28
no	yes	respiratory	yes	no		yes	no	3	3
no	no		no	no		no	yes	14	10
no	yes	other	no	no		no	yes	13	9
no	yes	cardiovascular	yes	no		yes	yes	4	2
no	no		no	no		no	no	9	9
no	yes	renal	yes	no		yes	yes	4	4
no	yes	other	no	no		no	yes	9	6
no	no		no	no		no	no	5	3
no	no		no	no		no	no	59	13
no	no		no	no		no	no	3	3
no	no		no	no		yes	yes	35	20
no	yes	cardiovascular	yes	no		no	no	3	3
no	yes	other	no	no		no	no	22	22
no	yes	other	no	no		no	yes	29	29
no	no		no	no		yes	yes	16	16
no	yes	hematologic	no	no		no	yes	13	11
no	yes	other	yes	no		yes	no	27	11
no	yes	other	no	no		no	no	4	4
yes	yes	other	no	yes	after intubation	no	no	29	29
no	yes	cardiovascular	yes	no		no	no	1	1
no	no		yes	no		yes	yes	26	22
yes	yes	other	no	no		no	no	1	1
no	no		no	no		no	no	20	5
no	no		no	no		no	yes	1	1
no	no		no	no		yes	yes	1	1
no	yes	respiratory	no	no		no	yes	4	4
no	no		no	no		no	no	6	6
no	yes	hepatic	no	no		no	no	15	5
no	yes	respiratory	no	no		no	yes	1	1
no	yes	other	no	no		yes	yes	16	13
yes	no		no	no		yes	yes	18	18
no	no		no	no		yes	yes	1	1
no	yes	other	no	no		yes	yes	3	3
no	yes	other	no	yes	after intubation	no	yes	30	30
no	no		no	yes	after intubation	no	no	70	37

no	no		no	no		no	yes	3	3
no	no		no	no		yes	yes	11	11
no	no		no	no		yes	yes	10	10
no	yes	cardiovascular	no	no		no	yes	6	6
no	yes	other	no	no		no	yes	12	12
no	no		no	no		no	no	9	9
yes	no		no	yes	after intubation	no	no	24	24
yes	yes	cardiovascular	no	no		yes	no	20	20
yes	yes	hepatic	no	no		yes	yes	10	10
yes	no		yes	no		no	no	18	18
yes	no		no	yes	after intubation	no	no	27	12
yes	yes	cardiovascular	yes	no		no	yes	28	5
yes	yes	cardiovascular	no	no		no	no	4	4
yes	no		yes	no		no	no	34	34
no	yes	other	no	no		yes	yes	15	15
no	yes	other	no	no		no	no	2	2
no	no		no	no		yes	yes	2	2
no	no		yes	no		yes	yes	5	3
yes	no		no	no		yes	yes	3	3
no	yes	hematologic	no	yes	after intubation	no	no	28	28
no	no		no	no		no	no	37	37
no	yes	cardiovascular	no	no		yes	yes	1	1
no	no		yes	no		no	no	1	1
no	no		no	no		yes	yes	29	9
no	yes	other	no	no		no	no	9	5
no	no		no	no		no	yes	9	9
no	no		yes	no		yes	yes	19	10
no	no		no	no		no	no	3	3
yes	yes	other	yes	no		no	yes	13	9
no	yes	respiratory	no	no		no	yes	2	2
no	no		no	no		yes	yes	10	10
no	no		yes	no		yes	yes	18	18
no	yes	other	no	no		no	no	15	15
no	no		no	no		yes	yes	1	1
no	yes	respiratory	no	no		no	yes	10	10
no	yes	respiratory	no	no		yes	yes	4	4
no	yes	cardiovascular	no	no		yes	yes	7	7
no	yes	other	yes	no		yes	yes	3	2
no	no		no	no		no	no	6	25
no	no		no	no		no	no	10	5
no	yes	other	no	no		no	yes	10	5
no	yes	other	no	no		no	no	5	1
no	no		no	no		no	no	14	14
no	yes	other	no	no		no	yes	49	44
no	yes	cardiovascular	no	no		no	yes	28	23
no	yes	other	no	no		no	no	3	3
no	no		no	no		no	no	14	3

no	no		no	no		no	yes	5	5
no	yes	other	yes	no		no	no	29	26
no	no		no	no		no	yes	10	10
no	no		no	no		yes	yes	4	4
no	yes	cardiovascular	yes	no		no	yes	5	5
no	yes	cardiovascular	yes	no		no	yes	8	8
no	no		yes	no		yes	yes	24	18
no	no		no	no		no	no	2	2
no	yes	other	yes	no		yes	yes	3	3
no	no		no	yes	after intubation	no	no	41	41
no	no		no	yes	after intubation	no	no	27	27
no	yes	cardiovascular	no	no		no	no	28	10
no	yes	cardiovascular	yes	no		no	no	1	1
no	yes	renal	no	no		no	yes	1	1
no	yes	other	yes	no		yes	yes	19	9
no	no		no	no		no	no	29	23

CMV	CPR	mode	LSMV	sepsis	dialysis	comp	outcome	plt	Cr
neurological	no	A/C	6	yes	no	no	transfer	267	1.06
neurological	no	A/C	6	yes	no	no	transfer	267	1.06
e respiratory fa	no	A/C	4	no	no	no	discharge	422	0.63
neuromuscular	yes	A/C	24	yes	no	no	death	186	0.95
e respiratory fa	no	A/C	3	no	no	no	transfer	451	1.08
neurological	no	A/C	13	no	no	no	LAMT	31	0.83
other	no	A/C	9	no	no	no	death	93	2.48
other	no	A/C	3	no	no	no	death	113	1.68
neuromuscular	no	A/C	23	yes	no	yes	death	160	1.8
cardiovascular	yes	SIMV	24	yes	no	yes	death	200	1.4
e respiratory fa	no	SIMV	3	no	no	no	LAMT	150	0.6
neuromuscular	no	SIMV	10	no	no	no	death	154	1.23
hematological	no	SIMV	5	no	no	no	death	148	12.3
e respiratory fa	no	A/C	8	yes	no	no	death	299	0.58
e respiratory fa	yes	A/C	2	no	no	no	death	191	1.78
other	no	A/C	2	no	no	no	transfer	154	0.49
e respiratory fa	no	A/C	2	no	no	no	transfer	326	0.83
neurological	no	A/C	3	no	no	no	death	524	2.03
e respiratory fa	no	A/C	4	yes	no	no	death	172	0.96
neurological	yes	A/C	5	no	no	no	death	85	1.35
e respiratory fa	no	SIMV	3	no	no	no	LAMT	170	1.14
e respiratory fa	no	SIMV	10	yes	no	no	death	197	0.88
e respiratory fa	yes	A/C	3	no	no	no	death	222	0.56
e respiratory fa	no	SIMV	14	yes	no	yes	reffered	169	0.75
hematological	no	A/C	3	no	no	no	death	37	2.12
neurological	no	A/C	19	yes	no	no	transfer	209	0.72
e respiratory fa	no	SIMV	24	yes	no	yes	LAMT	4	0.44
e respiratory fa	no	SIMV	12	yes	no	no	discharge	192	4.82
hematological	yes	A/C	11	no	no	no	death	408	0.72
e respiratory fa	no	A/C	25	yes	no	yes	death	161	0.82
neurological	yes	A/C	11	yes	no	no	death	23	0.5
neurological	yes	A/C	4	no	no	no	death	326	2.78
hematological	no	A/C	23	yes	no	yes	death	240	0.11
neurological	yes	A/C	1	no	no	no	death	163	0.43
neuromuscular	yes	A/C	3	no	no	no	death	150	0.79
e respiratory fa	yes	A/C	22	yes	no	yes	death	140	4.52
neurological	no	A/C	1	no	no	no	reffered	582	0.85
e respiratory fa	no	A/C	5	yes	no	no	LAMT	124	1.05
e respiratory fa	no	A/C	1	no	no	no	transfer	113	0.97
e respiratory fa	yes	A/C	1	no	no	no	death	137	2.47
e respiratory fa	yes	A/C	1	no	no	no	death	472	0.99
e respiratory fa	yes	A/C	2	no	no	no	transfer	89	1.1
e respiratory fa	no	A/C	4	no	no	no	transfer	298	1.08
neuromuscular	no	A/C	5	no	no	no	transfer	341	0.47
e respiratory fa	no	A/C	3	no	no	no	transfer	306	0.99
e respiratory fa	yes	A/C	1	no	no	no	death	153	1.45

e respiratory fa	yes	A/C	13	no	no	no	death	188	0.72
e respiratory fa	no	A/C	12	no	no	no	transfer	649	0.76
e respiratory fa	yes	A/C	1	no	no	no	death	289	0.88
e respiratory fa	yes	A/C	3	no	no	no	death	261	1.02
neuromuscular	no	A/C	22	yes	no	yes	death	469	0.65
other	no	A/C	25	yes	no	yes	death	441	0.59
neurological	yes	A/C	3	no	no	no	death	304	0.65
e respiratory fa	yes	A/C	4	no	no	no	death	198	2.98
e respiratory fa	no	A/C	6	no	no	no	transfer	243	0.93
e respiratory fa	no	A/C	5	no	no	no	transfer	258	1.11
neurological	no	A/C	5	no	no	no	transfer	378	3.03
e respiratory fa	yes	A/C	12	no	no	no	death	365	1.123
e respiratory fa	yes	A/C	4	yes	no	no	death	153	1.11
other	no	SIMV	3	yes	no	no	transfer	260	0.5
e respiratory fa	no	A/C	9	no	no	no	discharge	88	1.05
neuromuscular	yes	CPAP	4	no	no	no	death	224	1
neurological	no	A/C	5	yes	no	no	discharge	148	1.38
neurological	yes	A/C	2	no	no	no	death	290	2.09
e respiratory fa	no	A/C	10	no	no	no	transfer	437	0.7
neurological	no	A/C	16	no	no	no	LAMT	253	1.23
neuromuscular	yes	A/C	3	no	no	yes	death	50	0.79
neurological	yes	A/C	3	no	no	no	death	281	1.32
neuromuscular	no	A/C	12	no	no	yes	death	347	0.9
renal	no	A/C	11	no	no	no	discharge	380	1
neuromuscular	no	A/C	6	yes	no	no	transfer	559	0.71
e respiratory fa	no	A/C	5	yes	no	yes	death	249	1.24
e respiratory fa	yes	A/C	4	no	no	no	death	170	3.2
neuromuscular	no	A/C	2	no	no	no	death	217	1.13
e respiratory fa	no	A/C	27	yes	no	yes	death	99	1.18
e respiratory fa	no	A/C	4	no	no	no	transfer	104	0.6
e respiratory fa	no	A/C	15	no	no	no	LAMT	232	2.4
neurological	no	A/C	2	no	no	no	transfer	316	1.03
e respiratory fa	no	A/C	7	no	no	no	death	135	4.22
e respiratory fa	yes	A/C	2	no	no	no	death	256	1.4
e respiratory fa	yes	A/C	3	yes	no	no	death	85	3.96
e respiratory fa	yes	A/C	3	no	no	no	death	122	1.33
neurological	no	A/C	24	yes	no	yes	death	310	0.78
neurological	no	A/C	36	yes	no	yes	death	351	2.55
e respiratory fa	no	A/C	1	no	no	no	death	362	2.85
e respiratory fa	yes	A/C	1	no	no	no	death	323	1.97
e respiratory fa	yes	A/C	1	no	no	no	death	187	2.24
e respiratory fa	no	A/C	9	no	no	no	LAMT	261	0.7
e respiratory fa	no	A/C	5	no	no	no	death	93	2.21
e respiratory fa	no	SIMV	3	no	no	no	transfer	129	2.7
neurological	no	A/C	3	no	no	no	discharge	88	0.83
e respiratory fa	no	A/C	4	no	no	no	transfer	16	0.53
e respiratory fa	yes	A/C	3	no	no	no	death	183	0.98

e respiratory fa	yes	A/C	2	yes	no	no	death	213	1.22
e respiratory fa	yes	A/C	2	no	no	no	death	345	1
e respiratory fa	yes	A/C	6	no	no	no	death	21	2.59
neurological	no	SIMV	2	no	no	no	discharge	149	0.49
e respiratory fa	yes	A/C	9	no	no	no	death	195	0.83
e respiratory fa	no	SIMV	5	no	no	no	transfer	152	0.37
e respiratory fa	yes	A/C	2	no	no	no	death	186	1.03
e respiratory fa	no	SIMV	10	yes	no	no	LAMT	380	3.32
e respiratory fa	yes	A/C	18	yes	no	no	death	335	0.65
e respiratory fa	yes	A/C	1	yes	no	no	death	111	1.9
e respiratory fa	yes	A/C	3	yes	no	no	death	242	3.94
e respiratory fa	no	A/C	3	no	no	no	death	239	1.41
e respiratory fa	yes	A/C	15	yes	no	yes	death	227	0.5
e respiratory fa	yes	A/C	1	no	no	no	death	230	0.94
neurological	no	A/C	8	no	no	no	transfer	173	1.3
neuromuscular	yes	A/C	9	yes	no	no	death	405	1.7
e respiratory fa	yes	A/C	6	yes	no	no	death	255	1.1
e respiratory fa	no	A/C	3	no	no	no	transfer	177	1.24
neurological	yes	A/C	5	no	no	no	death	159	2.35
e respiratory fa	yes	A/C	4	no	no	no	death	84	1.23
e respiratory fa	yes	A/C	7	no	no	no	death	154	2.02
neurological	no	SIMV	2	no	no	no	discharge	279	1.12
e respiratory fa	no	A/C	7	no	no	no	LAMT	202	0.97
neuromuscular	yes	A/C	18	yes	no	yes	death	133	0.88
e respiratory fa	yes	A/C	2	no	no	no	death	150	1.17
e respiratory fa	no	A/C	12	no	no	no	transfer	253	1.23
hematological	yes	A/C	5	no	no	yes	death	131	5.7
e respiratory fa	no	A/C	23	yes	no	yes	transfer	670	0.79
e respiratory fa	no	A/C	3	no	no	no	transfer	200	0.9
e respiratory fa	yes	A/C	4	no	no	no	death	52	6.62
e respiratory fa	yes	A/C	8	yes	no	yes	death	188	1.06
e respiratory fa	yes	A/C	1	no	no	no	death	362	0.49
hematological	yes	A/C	2	no	no	no	discharge	26	1.63
e respiratory fa	no	A/C	16	yes	no	no	transfer	161	1.04
e respiratory fa	no	A/C	2	yes	no	yes	transfer	143	0.64
e respiratory fa	yes	A/C	16	yes	no	yes	death	256	0.97
neurological	no	A/C	5	no	no	no	discharge	192	1.15
e respiratory fa	yes	A/C	7	yes	no	no	death	97	1.38
neurological	yes	A/C	5	yes	no	no	death	85	0.79
e respiratory fa	no	A/C	10	yes	no	no	transfer	99	0.84
hematological	yes	A/C	1	no	no	no	death	108	0.84
e respiratory fa	no	A/C	2	no	no	no	transfer	119	0.63
e respiratory fa	no	A/C	2	no	no	no	death	138	1.89
e respiratory fa	no	A/C	14	yes	no	no	LAMT	188	0.62
cardiovascular	no	A/C	8	yes	no	no	LAMT	128	2.03
e respiratory fa	no	A/C	1	no	no	no	LAMT	181	0.64
cardiovascular	no	A/C	3	yes	no	no	transfer	159	0.55

e respiratory fa	yes	A/C	9	no	no	no	death	257	3.19
e respiratory fa	no	A/C	27	yes	no	yes	death	396	0.34
e respiratory fa	yes	A/C	3	no	no	no	death	85	0.28
e respiratory fa	no	A/C	4	no	no	no	transfer	696	0.4
e respiratory fa	yes	A/C	2	no	no	no	death	156	0.46
hematological	yes	A/C	9	no	yes	no	death	5	5.29
e respiratory fa	no	A/C	23	yes	no	yes	transfer	240	2.54
e respiratory fa	yes	A/C	3	no	no	no	death	303	2.17
e respiratory fa	yes	A/C	1	no	no	no	death	295	1.44
e respiratory fa	yes	A/C	2	no	no	no	death	276	1.02
e respiratory fa	no	A/C	3	yes	no	no	transfer	490	2.5
neurological	no	A/C	5	no	no	no	death	173	0.65
e respiratory fa	no	SIMV	2	no	no	no	discharge	154	0.38
e respiratory fa	no	A/C	27	yes	no	yes	transfer	299	
e respiratory fa	no	A/C	12	no	no	no	death	214	1.14
neuromuscular	no	A/C	26	yes	no	no	death	107	0.64
e respiratory fa	no	A/C	9	no	no	no	discharge	147	0.52
e respiratory fa	yes	A/C	6	no	no	no	death	128	1.4
e respiratory fa	yes	A/C	3	no	no	no	death	150	1.09
renal	no	A/C	10	no	yes	no	LAMT	133	7.9
e respiratory fa	yes	A/C	1	no	no	no	death	97	0.2
neurological	no	SIMV	5	no	no	no	discharge	88	0.4
e respiratory fa	yes	A/C	1	no	no	no	death	114	2.2
e respiratory fa	no	A/C	2	no	no	no	reffered	189	0.99
e respiratory fa	no	A/C	3	no	no	no	discharge	174	0.76
e respiratory fa	no	A/C	11	no	no	no	LAMT	470	0.74
e respiratory fa	yes	A/C	1	no	no	no	death	100	1.83
neurological	yes	A/C	3	no	no	no	death	171	0.99
cardiovascular	yes	A/C	5	no	yes	no	death	216	1.51
e respiratory fa	no	A/C	5	yes	no	no	transfer	133	1.02
e respiratory fa	yes	A/C	3	no	yes	no	death	290	7.66
e respiratory fa	yes	A/C	18	no	no	no	death	372	0.74
e respiratory fa	yes	A/C	9	no	no	no	death	92	1.71
e respiratory fa	yes	A/C	1	no	no	no	death	64	0.66
e respiratory fa	yes	A/C	1	no	no	no	death	366	2.24
e respiratory fa	yes	A/C	3	no	no	no	death	297	1
e respiratory fa	yes	A/C	23	yes	no	no	reffered	148	0.87
e respiratory fa	no	A/C	16	yes	no	no	death	444	0.94
e respiratory fa	no	A/C	23	yes	no	no	transfer	194	0.53
e respiratory fa	no	A/C	10	no	no	no	transfer	257	0.73
e respiratory fa	no	A/C	18	yes	no	no	discharge	155	0.55
e respiratory fa	yes	A/C	11	no	yes	no	death	180	2.11
e respiratory fa	yes	A/C	1	no	no	no	death	148	0.86
hematological	yes	A/C	1	no	no	no	death	164	3.15
neurological	yes	A/C	1	no	no	no	death	128	0.59
e respiratory fa	yes	A/C	1	no	no	no	death	276	2.3
neurological	yes	A/C	1	no	no	no	death	240	0.58

e respiratory fa	yes	A/C	9	yes	no	no	death	258	0.95
e respiratory fa	no	A/C	2	no	yes	no	reffered	208	4.83
neurological	yes	A/C	6	no	no	no	death	193	4.41
e respiratory fa	yes	A/C	6	no	no	no	death	163	0.77
e respiratory fa	no	A/C	23	yes	no	no	transfer	198	1.37
e respiratory fa	no	A/C	7	no	no	no	discharge	155	0.65
e respiratory fa	yes	A/C	9	yes	no	no	death	103	2.15
neurological	no	A/C	15	yes	no	no	discharge	387	0.48
e respiratory fa	no	SIMV	3	no	no	no	discharge	318	0.9
e respiratory fa	yes	A/C	1	no	no	no	death	274	1.23
neurological	yes	A/C	1	no	no	no	death	203	4.5
e respiratory fa	no	A/C	5	no	no	no	discharge	105	0.66
neurological	no	A/C	24	yes	yes	yes	death	164	0.44
e respiratory fa	yes	A/C	25	yes	yes	no	death	156	0.47
e respiratory fa	yes	A/C	7	no	no	no	death	128	0.85
cardiovascular	no	A/C	12	yes	yes	no	death	139	8.05
neurological	no	A/C	24	yes	no	yes	death	117	0.88
e respiratory fa	yes	A/C	18	no	yes	no	death	129	9.03
e respiratory fa	no	SIMV	7	no	no	no	transfer	135	0.59
e respiratory fa	no	A/C	24	yes	no	no	death	304	0.23
cardiovascular	yes	A/C	22	yes	yes	no	death	384	0.78
e respiratory fa	no	A/C	25	yes	no	no	death	374	0.78
e respiratory fa	no	A/C	15	yes	no	no	transfer	360	1.05
e respiratory fa	yes	A/C	9	no	no	no	death	340	1.09
hematological	no	SIMV	5	no	no	no	discharge	158	1.03
neuromuscular	yes	A/C	23	yes	no	yes	death	151	0.55
neuromuscular	yes	A/C	26	yes	no	no	death	142	1.01
neurological	no	A/C	24	yes	no	no	death	137	0.92
e respiratory fa	yes	A/C	22	yes	yes	no	death	191	5.89
e respiratory fa	no	A/C	22	yes	no	no	transfer	204	0.32
neurological	no	A/C	29	no	yes	no	transfer	222	0.33
e respiratory fa	no	A/C	29	yes	no	yes	transfer	389	0.67
e respiratory fa	no	A/C	24	yes	no	no	death	363	1.05
neuromuscular	yes	A/C	25	yes	no	no	death	378	0.55
neurological	no	A/C	13	yes	no	no	transfer	325	0.23
neurological	yes	A/C	23	no	no	no	death	330	0.88
neurological	no	A/C	18	no	no	no	death	322	0.93
e respiratory fa	yes	SIMV	9	no	no	no	death	350	1.03
e respiratory fa	no	A/C	23	yes	no	no	transfer	294	0.55
neurological	no	A/C	30	yes	no	yes	death	335	0.62
e respiratory fa	yes	A/C	3	no	no	no	death	255	0.28
neuromuscular	no	A/C	23	yes	no	no	death	263	0.45
neurological	no	SIMV	26	yes	no	no	transfer	240	0.89
neuromuscular	yes	SIMV	23	yes	no	no	death	220	0.74
e respiratory fa	no	A/C	3	no	no	no	discharge	187	0.65
e respiratory fa	no	A/C	6	no	no	no	transfer	163	0.84
neurological	yes	A/C	24	yes	no	no	death	146	0.33

e respiratory fa	no	A/C	22	yes	no	no	transfer	194	0.62
neurological	yes	A/C	23	yes	no	yes	death	360	0.48
neurological	yes	A/C	27	yes	no	yes	death	335	1.02
neuromuscular	no	A/C	27	yes	no	yes	transfer	363	0.84
e respiratory fa	yes	A/C	23	yes	yes	no	death	340	0.49
neurological	no	A/C	28	yes	no	yes	death	266	0.841
neurological	no	A/C	4	no	no	no	death	259	0.77
e respiratory fa	yes	A/C	1	no	no	no	death	274	1.23
neurological	yes	A/C	1	no	no	no	death	203	4.5
e respiratory fa	no	A/C	5	no	no	no	discharge	104	0.61
neurological	no	A/C	24	yes	yes	yes	death	166	0.54
e respiratory fa	yes	A/C	25	yes	yes	no	death	146	0.37
e respiratory fa	yes	A/C	7	no	no	no	death	138	0.75
cardiovascular	no	A/C	12	yes	yes	no	death	149	9
neurological	no	A/C	24	yes	no	yes	death	119	0.77
e respiratory fa	yes	A/C	18	no	yes	no	death	324	8.74
e respiratory fa	no	SIMV	7	no	no	no	transfer	137	0.69
e respiratory fa	no	A/C	24	yes	no	no	death	324	0.28
cardiovascular	yes	A/C	22	yes	yes	no	death	365	0.74
e respiratory fa	no	A/C	25	yes	no	no	death	362	0.87
e respiratory fa	no	A/C	15	yes	no	no	transfer	345	1.02
e respiratory fa	yes	A/C	9	no	no	no	death	331	1.01
hematological	no	SIMV	5	no	no	no	discharge	158	1.03
neuromuscular	yes	A/C	23	yes	no	yes	death	152	0.56
neuromuscular	yes	A/C	26	yes	no	no	death	148	1.01
neurological	no	A/C	24	yes	no	no	death	139	0.82
e respiratory fa	yes	A/C	22	yes	yes	no	death	191	5.89
e respiratory fa	no	A/C	22	yes	no	no	transfer	206	0.32
neurological	no	A/C	29	no	yes	no	transfer	222	0.33
e respiratory fa	no	A/C	29	yes	no	yes	transfer	389	0.67
e respiratory fa	no	A/C	24	yes	no	no	death	365	1.06
neuromuscular	yes	A/C	25	yes	no	no	death	378	0.63
neurological	no	A/C	13	yes	no	no	transfer	325	0.23
neurological	yes	A/C	23	no	no	no	death	330	0.88
neurological	no	A/C	18	no	no	no	death	326	0.92
e respiratory fa	yes	SIMV	9	no	no	no	death	350	1.03
e respiratory fa	no	A/C	23	yes	no	no	transfer	294	0.53
neurological	no	A/C	30	yes	no	yes	death	335	0.62
e respiratory fa	yes	A/C	3	no	no	no	death	255	0.28
neuromuscular	no	A/C	23	yes	no	no	death	263	0.45
neurological	no	SIMV	26	yes	no	no	transfer	240	0.89
neuromuscular	yes	SIMV	23	yes	no	no	death	220	0.74
e respiratory fa	no	A/C	3	no	no	no	discharge	187	0.65
e respiratory fa	no	A/C	6	no	no	no	transfer	163	0.84
neurological	yes	A/C	24	yes	no	no	death	149	0.33
e respiratory fa	no	A/C	22	yes	no	no	transfer	194	0.62
neurological	no	A/C	23	yes	no	yes	death	360	0.48

neurological	yes	A/C	27	yes	no	yes	death	335	1.02
neuromuscular	no	A/C	27	yes	no	yes	transfer	363	0.84
e respiratory fa	yes	A/C	23	yes	yes	no	death	340	0.49
neurological	no	A/C	28	yes	no	yes	death	266	0.81
neurological	no	A/C	4	no	no	no	death	259	0.77
neurological	no	A/C	6	yes	no	no	transfer	267	1.06
neuromuscular	yes	A/C	24	yes	no	no	death	186	0.95
neurological	no	A/C	13	no	no	no	LAMT	31	0.83
other	no	A/C	9	no	no	no	death	93	2.48
other	yes	A/C	3	no	no	no	death	113	1.68
neuromuscular	no	A/C	23	yes	no	yes	death	160	1.8
cardiovascular	yes	SIMV	24	yes	no	yes	death	200	1.4
e respiratory fa	no	SIMV	3	no	no	no	LAMT	150	0.6
neuromuscular	no	SIMV	10	no	no	no	death	154	1.23
e respiratory fa	yes	A/C	8	yes	no	no	death	299	0.58
e respiratory fa	yes	A/C	2	no	no	no	death	191	1.78
e respiratory fa	no	A/C	2	no	no	no	transfer	154	0.49
neurological	yes	A/C	3	no	yes	no	death	524	2.03
e respiratory fa	no	A/C	4	yes	no	no	death	172	0.99
e respiratory fa	no	SIMV	3	no	no	no	LAMT	170	1.14
e respiratory fa	no	SIMV	10	yes	no	no	death	197	0.88
e respiratory fa	yes	A/C	3	no	no	no	death	222	0.56
e respiratory fa	no	SIMV	14	yes	no	yes	reffered	169	0.75
hematological	yes	A/C	3	no	no	no	death	37	2.12
neurological	no	A/C	19	yes	no	no	transfer	209	0.72
e respiratory fa	no	SIMV	24	yes	no	yes	LAMT	4	0.44
e respiratory fa	no	SIMV	12	yes	no	no	discharge	192	4.823
hematological	yes	A/C	11	no	no	no	death	408	0.72
neurological	yes	A/C	11	yes	no	no	death	23	0.5
neurological	yes	A/C	4	no	no	no	death	326	2.78
hematological	no	A/C	23	yes	no	yes	death	240	0.11
neurological	yes	A/C	1	no	no	no	death	163	0.43
e respiratory fa	yes	A/C	22	yes	yes	yes	death	140	4.52
neurological	no	A/C	1	no	no	no	reffered	582	0.85
e respiratory fa	no	A/C	5	yes	no	no	LAMT	124	1.05
e respiratory fa	no	A/C	1	no	no	no	transfer	113	0.97
e respiratory fa	yes	A/C	1	no	no	no	death	137	2.47
e respiratory fa	yes	A/C	1	no	no	no	death	472	0.91
e respiratory fa	no	A/C	4	no	no	no	transfer	298	1.08
neuromuscular	no	A/C	5	no	no	no	transfer	341	0.47
e respiratory fa	yes	A/C	1	no	no	no	death	153	2.45
e respiratory fa	yes	A/C	13	no	no	no	death	188	0.72
e respiratory fa	no	A/C	12	no	no	no	transfer	649	0.76
e respiratory fa	yes	A/C	1	no	no	no	death	289	0.88
e respiratory fa	yes	A/C	3	no	no	no	death	261	1.02
neuromuscular	no	A/C	22	yes	no	yes	death	469	0.65
other	no	A/C	25	yes	no	yes	death	441	0.59

neurological	yes	A/C	3	no	no	no	death	304	0.6
e respiratory fa	no	A/C	6	no	no	no	transfer	243	0.93
e respiratory fa	no	A/C	5	no	no	no	transfer	258	1.11
neurological	no	A/C	5	no	yes	no	transfer	378	3.03
e respiratory fa	no	A/C	9	no	no	no	discharge	88	1.05
neurological	no	A/C	5	yes	no	no	discharge	148	1.35
e respiratory fa	no	A/C	10	no	no	no	transfer	437	0.7
neurological	no	A/C	16	yes	no	no	LAMT	253	1.23
neuromuscular	yes	A/C	10	yes	no	yes	death	50	0.79
neuromuscular	no	A/C	12	no	no	yes	death	347	0.9
neuromuscular	no	A/C	6	yes	no	no	transfer	559	0.71
e respiratory fa	no	A/C	5	yes	no	yes	death	249	1.24
e respiratory fa	yes	A/C	4	no	no	no	death	170	3.2
e respiratory fa	no	A/C	27	yes	no	yes	death	99	1.18
e respiratory fa	no	A/C	15	no	no	no	LAMT	232	2.4
neurological	no	A/C	2	no	no	no	transfer	316	1.03
e respiratory fa	yes	A/C	2	no	no	no	death	250	1.4
e respiratory fa	yes	A/C	3	yes	no	no	death	85	3.96
e respiratory fa	yes	A/C	3	no	no	no	death	122	1.33
neurological	no	A/C	24	yes	no	yes	death	310	0.78
neurological	no	A/C	36	yes	no	yes	death	351	2.55
e respiratory fa	yes	A/C	1	no	no	no	death	323	1.97
e respiratory fa	yes	A/C	1	no	no	no	death	187	2.24
e respiratory fa	no	A/C	9	no	no	no	LAMT	261	0.7
e respiratory fa	no	A/C	5	no	no	no	death	93	2.21
neurological	no	A/C	3	no	no	no	discharge	88	0.83
e respiratory fa	yes	A/C	8	no	no	no	death	21	2.59
neurological	no	SIMV	2	no	no	no	discharge	149	0.49
e respiratory fa	yes	A/C	9	no	no	no	death	195	0.83
e respiratory fa	yes	A/C	1	no	no	no	death	186	1.03
e respiratory fa	no	SIMV	10	yes	no	no	LAMT	380	3.32
e respiratory fa	yes	A/C	18	yes	no	no	death	335	0.67
e respiratory fa	yes	A/C	15	yes	no	yes	death	227	0.5
e respiratory fa	yes	A/C	1	no	no	no	death	230	0.94
neurological	no	A/C	9	no	no	no	transfer	173	1.3
e respiratory fa	yes	A/C	4	no	no	no	death	84	1.23
e respiratory fa	yes	A/C	7	no	no	no	death	154	2.02
e respiratory fa	yes	A/C	2	no	no	no	death	150	1.17
e respiratory fa	no	A/C	23	yes	no	yes	transfer	670	0.79
neurological	no	A/C	5	no	no	no	discharge	192	1.15
neurological	yes	A/C	5	yes	no	no	death	85	0.79
hematological	yes	A/C	1	no	no	no	death	108	0.84
e respiratory fa	no	A/C	14	yes	no	no	LAMT	188	0.62
e respiratory fa	no	A/C	27	yes	no	yes	death	396	0.34
e respiratory fa	no	A/C	23	yes	no	yes	transfer	240	2.54
e respiratory fa	yes	A/C	3	no	no	no	death	303	2.17
e respiratory fa	no	A/C	3	yes	no	no	transfer	490	2.5

neurological	no	A/C	5	no	no	no	death	173	0.63
neuromuscular	no	A/C	26	yes	no	no	death	107	0.64
e respiratory fa	no	A/C	9	no	no	no	discharge	147	0.52
e respiratory fa	no	A/C	2	no	no	no	reffered	189	0.99
e respiratory fa	yes	A/C	5	no	yes	no	death	216	1.51
e respiratory fa	yes	A/C	3	no	yes	no	death	290	7.66
e respiratory fa	yes	A/C	18	no	no	no	death	372	0.74
e respiratory fa	yes	A/C	1	no	no	no	death	366	2.24
e respiratory fa	yes	A/C	3	no	no	no	death	297	1
e respiratory fa	yes	A/C	23	yes	no	no	reffered	148	0.87
e respiratory fa	no	A/C	23	yes	no	no	transfer	194	0.53
e respiratory fa	no	A/C	10	no	no	no	transfer	257	0.73
e respiratory fa	yes	A/C	1	no	no	no	death	148	0.86
hematological	yes	A/C	1	no	no	no	death	164	3.154
e respiratory fa	yes	A/C	9	yes	no	no	death	258	0.95
e respiratory fa	no	A/C	23	yes	no	no	transfer	198	1.37

temprature	SBp	DBp	HR	RR	FiO2	SpO2	GCScat	pnumoniacat	LScat
36.5	130	75	108	28	100	86	3	2	2
36.5	130	75	108	28	100	86	3	2	2
36	130	80	120	28	60	90	3	2	2
36.6	104	68	94	24	40	96	3	2	2
36.7	140	80	156	50	50	80	3	2	1
36	109	60	91	18	95	94	1	2	2
35.9	90	60	124	22	90	96	1	2	2
37.4	92	52	134	18	100	99	1	2	1
35	90	50	120	26	90	93	1	2	2
36.3	100	70	98	24	70	96	3	2	2
37	100	60	78	18	50	96	3	2	1
37.6	140	80	112	40	95	93	3	1	2
36.4	103	65	108	15	100	96	3	1	2
34.2	120	88	86	15	40	95	2	1	2
35	100	60	82	26	40	70	1	2	2
37.8	150	100	140	38	100	70	1	2	2
36.8	100	60	137	50	80	83	3	1	2
36.1	90	60	80	28	100	80	1	1	1
37	140	70	84	48	80	84	3	1	2
38.1	110	70	108	32	50	63	2	1	2
38.3	120	70	68	30	100	91	1	2	2
32	100	70	92	20	100	92	3	2	2
35	72	39	82	13	100	60	1	2	1
35.7	90	56	139	28	70	83	1	1	2
36.6	160	110	89	28	100	68	1	2	1
34	90	60	120	5	100	72	1	2	2
36	140	80	110	29	90	78	3	1	2
35.8	140	70	110	42	100	44	3	1	2
39.4	120	80	119	22	90	80	2	1	2
37.1	99	67	89	28	70	85	2	2	2
36.7	90	60	86	24	70	85	1	2	2
36.4	132	70	82	30	100	70	1	2	1
36	130	70	124	20	100	91	3	2	2
34.3	70	40	108	5	100	68	1	2	1
37.3	90	50	111	18	100	91	3	2	1
36.5	120	80	80	40	100	70	3	1	2
34.8	130	75	62	10	100	64	1	2	1
36.3	110	70	98	32	100	87	2	2	2
37	83	62	109	28	100	96	1	1	1
39.2	90	50	140	44	100	47	2	1	1
37.1	85	50	106	45	100	79	1	1	1
35.5	135	95	115	27	100	87	3	1	1
34	110	70	123	28	100	81	1	2	2
36.5	110	70	88	22	100	90	3	2	2
36	100	70	90	40	100	79	3	2	1
37.2	80	50	112	34	100	60	3	1	1

37.6	90	50	108	34	100	82	3	1	2
37.2	90	60	88	22	60	86	2	1	2
38.7	70	60	120	30	80	87	1	1	1
33	110	70	100	39	100	80	1	1	1
38	118	65	121	27	100	99	3	1	2
36.5	150	100	130	28	80	90	1	2	2
36.8	70	50	165	22	100	80	1	1	1
38.1	70	50	140	48	100	81	1	1	1
35.2	128	91	132	44	100	45	3	1	2
36.8	90	60	116	28	100	60	1	1	2
36.2	110	70	96	16	100	90	3	1	2
39	100	60	118	22	100	81	3	1	2
32	86	58	50	20	100	86	3	1	2
36.3	100	60	120	42	80	78	3	2	2
35	104	64	94	27	100	86	3	1	2
36.1	120	70	106	21	94	88	3	2	2
35.8	110	89	97	16	90	84	1	2	2
40.2	100	60	168	40	80	87	1	2	1
36.1	110	60	97	17	90	88	1	2	2
35.5	80	50	90	28	100	93	2	1	2
36	100	70	100	28	80	94	1	1	2
37.8	113	56	120	38	100	76	1	1	2
37.7	120	90	108	26	100	90	1	2	2
35.4	92	53	96	24	100	96	1	2	2
35.6	130	70	94	24	80	96	1	2	2
35.8	120	80	108	24	80	87	3	1	2
35.9	120	70	94	28	100	92	2	2	1
36.7	110	60	90	22	100	93	3	2	1
36.3	150	100	124	32	60	85	1	2	2
36	130	70	105	22	80	92	1	2	2
36	125	90	106	38	100	56	3	1	2
38.8	140	60	130	55	100	65	1	2	1
38.2	100	70	120	36	100	84	3	1	2
39	120	70	146	48	80	58	3	1	1
38.4	108	63	114	20	100	83	3	1	2
37.5	90	50	120	30	100	86	1	1	1
36.3	80	50	108	26	100	89	1	2	2
39	90	54	80	15	80	86	1	2	2
36	110	70	100	12	100	76	2	2	1
36.1	187	112	140	72	100	52	3	1	1
36.7	98	50	91	24	100	80	1	2	1
37	100	60	81	20	80	86	3	1	2
37.7	100	50	78	34	90	78	1	2	2
39	180	110	130	48	40	88	1	2	1
36.9	110	60	75	24	100	86	1	1	2
36.1	98	62	84	36	80	75	3	1	2
36.3	80	40	72	20	100	73	1	1	1

36.2	140	80	108	32	95	56	3	1	2
37	120	70	92	20	100	88	3	2	2
37	80	50	92	37	60	80	3	1	2
35.8	110	70	88	18	100	63	1	2	1
36.9	110	70	110	28	80	92	3	1	2
36	130	90	100	48	80	43	3	1	2
35.7	110	70	100	56	70	72	1	1	1
35	135	85	94	32	60	74	2	1	2
38.4	110	70	104	29	60	46	3	1	2
40	90	60	120	38	80	65	3	1	1
38	110	70	140	38	100	63	1	2	1
36	140	60	60	40	100	82	1	1	1
36	118	75	136	42	100	80	1	2	2
36	160	90	112	32	100	80	1	1	1
38.3	107	53	106	34	100	82	1	1	2
36.5	120	70	133	24	60	89	3	1	2
38.4	100	60	136	45	100	64	3	2	2
36	85	50	122	28	100	76	1	1	2
36.2	100	70	100	32	100	72	1	1	2
39	160	80	118	42	80	62	2	1	1
36	127	76	136	60	100	50	2	1	2
36.5	115	70	92	22	40	92	1	1	2
36.5	80	50	100	22	60	90	2	2	2
36.1	130	80	64	18	100	85	3	2	2
36.7	85	60	104	24	100	88	1	1	1
39	130	70	134	30	40	84	1	1	2
37.6	70	40	109	26	70	70	1	2	2
35	80	40	143	44	100	65	1	2	2
37	100	70	132	26	100	91	3	2	1
36.5	110	70	78	20	100	87	3	1	1
36.8	109	62	78	8	100	89	3	2	2
36	120	60	132	36	100	60	3	1	1
33.6	110	70	100	14	100	78	2	2	2
37.4	110	70	112	26	100	84	3	2	2
36.5	130	80	96	36	80	90	3	1	2
36.9	90	60	71	20	50	96	3	2	2
36.7	170	90	82	24	100	90	1	2	2
37.9	100	80	117	20	60	76	3	1	2
39.4	140	90	94	28	100	84	1	1	2
37.9	150	100	112	29	60	89	3	1	2
35.6	106	80	92	20	50	93	3	2	1
36.7	110	70	108	18	60	85	2	2	2
35.9	90	50	124	32	60	97	1	2	1
36.5	110	70	80	16	100	84	1	2	2
36.7	120	91	98	22	40	97	3	2	2
38.6	80	60	148	36	100	82	3	2	1
36.1	90	60	96	18	40	96	3	2	2

37	100	48	125	11	100	55	1	1	2
38.5	160	80	105	27	70	86	2	1	2
39	82	62	140	32	100	88	3	2	1
37.4	102	58	100	16	80	95	3	1	2
36	90	60	140	36	60	88	2	1	1
36.5	106	71	83	12	100	70	1	1	2
37	160	95	70	18	100	90	1	1	2
36.2	140	90	64	22	80	87	1	2	1
36	100	60	89	20	80	94	3	1	1
35.8	80	50	100	24	50	91	1	2	1
38	84	60	70	26	80	94	2	2	2
36	130	70	40	22	40	94	1	1	2
37	139	86	140	38	40	84	3	1	1
36.5	157	89	129	22	50	92	2	2	2
36	110	70	100	28	80	65	3	2	2
37	100	70	101	24	50	92	3	2	2
37.4	100	70	134	24	40	82	1	1	2
36	90	60	123	22	50	92	3	2	2
35.9	83	56	90	20	60	90	3	1	1
37.2	180	140	73	34	80	70	3	2	2
38.7	80	40	115	31	100	72	3	2	1
35.9	100	70	86	22	40	96	3	2	2
37.8	104	64	100	34	60	83	3	2	1
36.1	100	70	91	36	60	71	3	1	1
37.8	110	60	97	23	40	94	3	2	1
36.8	103	77	112	50	60	80	2	1	2
39.3	80	50	155	30	50	90	2	2	1
38	96	58	104	48	80	88	1	2	2
36.5	138	74	100	21	40	93	3	1	2
36.4	100	60	60	22	40	74	1	1	2
37.4	90	50	78	28	50	79	3	1	2
35.3	106	70	70	20	50	92	2	1	2
36.8	116	83	86	22	60	94	1	2	2
37	75	47	129	35	80	82	1	1	1
34	110	70	100	42	100	80	3	2	1
36.8	140	120	137	28	100	56	3	1	1
37.4	100	60	120	28	60	92	1	2	2
36.4	118	70	150	32	40	93	3	2	2
35.8	119	68	91	22	40	92	2	2	2
36	109	60	86	28	60	79	1	2	2
35.9	120	80	130	24	50	90	3	1	2
36	130	70	120	18	60	92	2	1	2
38	198	140	120	12	80	60	2	2	1
37.2	120	80	112	18	60	91	1	1	1
38.3	140	60	80	68	100	80	1	2	1
36	120	80	92	36	100	80	1	1	1
36.5	110	70	88	38	60	85	1	1	1

38.6	100	60	150	40	80	70	3	1	2
39.4	140	100	100	42	60	82	3	1	1
39.2	100	70	90	10	100	48	1	1	2
40	120	80	100	24	80	92	3	2	2
37.5	113	73	98	20	60	84	3	2	2
36.3	110	60	105	18	40	89	1	2	2
36.2	130	80	86	28	50	87	3	2	2
37.4	137	89	132	32	40	98	2	2	2
36.3	119	77	100	30	60	84	2	1	2
36	110	70	100	40	100	40	3	2	1
34	110	70	100	41	100	63	1	2	1
36.3	100	58	78	22	80	75	1	2	2
36	110	70	84	20	60	80	1	2	2
36.3	110	60	90	18	40	86	1	2	2
39.2	128	72	74	42	100	58	3	1	2
37	130	70	88	30	80	62	3	1	2
36	127	67	76	12	60	81	1	2	2
35.8	115	78	80	20	60	80	3	1	2
36.7	112	68	79	22	40	87	1	2	2
36.8	130	70	86	20	40	84	3	2	2
36.4	125	76	94	22	50	76	3	2	2
40.2	140	90	66	38	70	63	3	1	2
39.3	100	70	78	34	60	77	3	1	2
37	112	68	90	22	50	80	1	2	2
37.1	128	63	86	20	40	90	2	2	2
37.8	116	65	82	16	60	89	1	2	2
36.7	123	74	78	20	40	84	3	2	2
35.7	190	145	103	6	100	65	1	2	2
36	140	80	76	18	60	84	1	2	2
32.8	129	75	68	12	80	83	2	1	2
35.7	205	185	118	18	70	85	3	2	2
36.7	140	88	122	20	40	84	3	2	2
35.6	128	62	109	22	100	75	1	2	2
37.8	130	70	120	20	100	90	3	2	2
35.6	115	78	100	22	70	62	1	1	2
37.2	190	83	118	8	100	58	1	2	2
36	180	115	130	12	100	74	1	2	2
34.3	118	62	128	18	60	80	1	2	2
33.8	80	50	134	16	80	83	1	2	2
37.5	119	70	110	13	80	90	1	1	2
36	140	100	115	22	60	81	1	2	1
35.3	110	73	120	22	50	86	3	2	2
36.3	125	67	132	18	80	80	1	2	2
35.5	130	80	114	20	60	78	2	2	2
34.8	80	50	110	12	100	63	1	1	2
35.7	140	90	84	20	80	88	3	1	2
35.9	135	78	90	18	60	80	2	1	2

36.5	132	80	80	22	100	58	1	2	2
37	180	120	136	10	100	66	1	2	2
36.8	209	184	140	12	80	86	1	1	2
36	130	90	136	18	60	83	1	2	2
37	128	62	104	14	80	83	1	2	2
36.8	190	154	142	16	100	78	1	2	2
36.6	202	185	128	20	100	74	1	2	1
36	110	70	100	40	100	40	3	2	1
34	110	70	100	41	100	63	1	2	1
36.3	100	58	78	22	80	75	1	2	2
37	110	80	86	20	60	80	1	2	2
36.4	110	60	90	18	40	86	1	2	2
39	128	72	74	42	100	58	3	1	2
37	130	80	82	32	80	62	3	1	2
36	120	67	78	12	60	81	1	2	2
35	115	74	80	20	60	80	3	1	2
36.7	112	69	82	22	40	87	1	2	2
36.9	130	70	84	20	40	84	3	2	2
36.2	125	76	94	22	50	76	3	2	2
39.3	140	90	76	38	70	63	3	1	2
38.9	100	70	78	34	60	81	3	1	2
36.2	116	71	90	22	50	80	1	2	2
37.1	128	63	86	20	40	90	2	2	2
37.8	116	65	82	16	60	89	1	2	2
36.7	123	74	78	20	40	84	3	2	2
35.7	190	145	103	6	100	65	1	2	2
36	140	80	76	18	60	84	1	2	2
32.8	129	75	68	12	80	83	2	1	2
35.7	205	185	118	18	70	85	3	2	2
36.7	140	88	122	20	40	84	3	2	2
35.3	128	62	109	22	100	75	1	2	2
37.8	130	70	120	20	100	90	3	2	2
35.6	115	78	100	22	70	62	1	1	2
37.2	190	83	118	8	100	58	1	2	2
36	180	115	130	12	100	74	1	2	2
34.3	118	62	128	18	60	80	1	2	2
33.8	80	50	134	16	80	83	1	2	2
37.5	119	78	110	13	80	90	1	1	2
36	140	100	115	22	60	81	1	2	1
35.2	110	73	120	22	50	86	3	2	2
36.3	125	67	132	18	80	80	1	2	2
35.5	130	80	114	20	60	78	2	2	2
34.8	80	50	110	12	100	63	1	1	2
35.7	140	90	84	20	80	88	3	1	2
35.9	135	78	90	18	60	80	2	1	2
36.5	132	80	80	22	100	58	1	2	2
37	180	120	136	10	100	66	1	2	2

36.8	209	184	140	12	80	86	1	1	2
36	130	90	136	18	60	83	1	2	2
37	128	62	104	14	80	83	2	2	2
36.8	190	157	142	16	100	78	1	2	2
36.6	202	185	128	20	100	74	1	2	1
36.5	130	75	108	28	100	86	3	2	2
36.6	104	68	94	24	40	96	3	2	2
36	109	60	91	18	95	94	1	2	2
35.9	90	60	124	22	90	96	1	2	2
37.4	92	52	134	18	100	99	1	2	1
35	90	50	120	26	90	93	1	2	2
36.3	100	70	98	24	70	96	3	2	2
37	100	60	78	18	50	90	3	2	1
37.6	140	80	112	40	95	93	3	1	2
34.2	129	88	86	15	40	95	2	1	2
35	100	60	82	26	40	70	3	1	1
37.8	150	100	140	38	100	70	1	2	2
36.1	90	60	80	28	100	80	1	1	1
37	140	70	84	48	80	84	3	1	2
38.3	120	70	68	30	100	91	1	2	2
37	100	70	92	20	100	92	3	2	2
35	72	39	82	13	100	60	1	2	1
35.7	90	56	139	28	70	83	1	1	2
36.6	160	110	89	28	100	68	1	2	1
34	90	60	120	5	100	72	1	2	2
36	140	80	110	29	90	78	3	1	2
35.8	140	70	110	42	100	40	3	1	2
39.4	120	80	119	22	90	80	2	1	2
36.7	90	60	86	24	70	85	3	2	2
36.4	132	70	82	30	100	70	1	2	1
36	130	70	124	20	100	91	3	2	2
34.3	70	40	108	5	100	68	1	2	1
36.5	120	80	80	40	100	70	3	1	2
34.8	130	75	62	10	100	64	1	2	1
36.3	110	70	98	32	100	87	2	2	2
37	83	62	109	28	100	96	1	1	1
39.2	90	50	140	44	100	47	2	1	1
37.1	85	50	106	45	100	79	3	1	1
34	110	70	123	28	100	81	1	2	2
36.5	110	60	88	22	100	90	3	2	2
37.2	80	50	112	34	100	60	3	1	1
37.6	90	50	108	34	100	82	3	1	2
37.2	90	60	92	32	60	86	1	1	2
38.7	90	60	120	30	80	87	1	1	1
33	110	70	100	39	100	80	1	1	1
38	118	65	121	27	100	91	1	1	2
36.5	150	100	130	28	80	90	1	2	2

36.8	70	50	165	22	100	80	1	1	1
35.2	128	91	132	44	100	45	3	1	2
36.8	90	60	116	28	100	60	1	1	2
36.2	110	70	96	16	100	90	3	1	2
35	104	64	94	27	100	86	3	1	2
35.8	110	89	97	16	90	94	1	2	2
36.1	110	60	97	17	90	85	1	2	2
35.5	80	50	90	28	100	93	2	2	2
36	100	70	100	28	80	94	1	1	2
37.7	120	90	108	26	100	90	1	2	2
35.6	130	70	94	24	80	96	1	2	2
35.8	120	80	108	24	80	87	3	1	2
35.9	120	70	94	28	100	92	2	2	1
36.6	150	100	124	32	60	85	1	2	2
36	125	90	106	38	100	56	3	1	2
38.8	140	60	130	55	100	65	1	2	1
39	120	70	146	48	100	58	3	1	1
38.4	108	63	114	20	100	83	3	1	2
37.5	90	50	120	30	100	86	1	1	1
36.3	80	50	108	26	100	89	1	2	2
39	90	54	80	15	80	86	1	2	2
36.1	187	112	140	72	100	52	3	1	1
36.7	98	50	91	24	100	80	1	2	1
37	100	60	81	20	80	86	3	1	2
37.7	100	50	78	34	90	78	1	2	2
36.9	110	60	75	24	100	86	1	1	2
37	80	50	92	38	60	80	3	1	2
35.8	110	70	88	18	100	63	1	2	1
36.9	110	70	110	28	80	92	3	1	2
35.7	110	70	100	56	70	72	1	1	1
35	135	85	94	32	60	74	2	1	2
38.4	135	85	104	38	100	46	1	1	2
36	118	71	136	42	100	80	1	2	2
36	160	90	112	32	100	80	1	1	1
38.3	107	53	106	34	100	82	1	1	2
39	160	80	118	42	80	62	2	1	1
36	127	76	136	60	100	50	2	1	2
36.7	85	60	104	24	100	88	1	1	1
35	80	40	143	44	100	65	1	2	2
36.7	170	90	82	24	100	90	1	2	2
39.4	140	90	94	28	100	84	1	1	2
35.6	106	80	92	20	50	93	3	2	2
36.5	110	70	80	16	100	84	1	2	2
38.5	160	80	105	27	70	86	2	1	2
37	160	95	70	18	100	90	1	1	2
36.2	140	90	64	22	80	87	1	2	1
38	84	60	70	26	80	94	2	2	2

36	130	70	40	22	40	94	1	1	2
37	100	70	101	24	50	92	3	2	2
37.4	100	70	134	24	40	82	1	1	2
36.1	100	70	91	36	60	71	3	1	1
36.5	138	74	100	21	40	93	3	1	2
37.4	90	50	78	28	50	79	3	1	2
35.3	106	70	70	20	50	92	2	1	2
34	110	70	100	42	100	80	3	2	1
36.8	140	120	137	28	100	56	3	1	1
37.4	100	60	120	28	60	92	1	2	2
35.8	119	68	91	22	40	92	2	2	2
36	109	69	86	28	60	79	1	2	2
38	198	140	120	12	80	60	2	2	1
37.2	120	80	112	18	60	91	1	1	1
38.6	100	60	100	40	80	70	1	1	2
37.5	113	73	98	20	60	84	3	2	2

Outcome	HRcat	SpO2cat	SBpcat	tempraturecat	pltcat	Agecat1	Dxcat	GCScat1	ICUWcat1
0	3	1	2	2	4	4	6	3	3
0	3	1	2	2	4	4	6	3	3
0	3	2	2	2	4	1	1	3	3
1	2	2	2	2	4	1	9	3	3
0	3	1	3	2	4	1	1	3	2
0	2	2	2	2	1	1	6	1	3
1	3	2	2	2	2	3	3	1	3
1	3	2	2	2	3	1	7	1	1
1	3	2	2	1	4	3	9	1	3
1	2	2	2	2	4	3	9	3	3
1	2	2	2	2	4	2	5	3	1
1	3	2	3	2	4	4	9	3	3
1	3	2	2	2	3	1	5	3	2
1	2	2	2	1	4	2	3	2	3
1	2	1	2	1	4	2	6	1	3
0	3	1	3	2	4	1	6	1	1
0	3	1	2	2	4	1	1	3	2
1	2	1	2	2	4	1	1	1	2
1	2	1	3	2	4	3	9	3	2
1	3	1	2	3	2	1	1	2	2
0	2	2	2	3	4	1	6	1	1
1	2	2	2	1	4	1	7	3	3
1	2	1	1	1	4	1	8	1	1
0	3	1	2	2	4	1	1	1	3
1	2	1	3	2	1	1	6	1	1
0	3	1	2	1	4	1	6	1	3
0	3	1	3	2	1	2	9	3	3
0	3	1	3	2	4	3	1	3	3
1	3	1	2	3	4	1	9	2	3
1	2	1	2	2	4	1	8	2	3
1	2	1	2	2	1	2	1	1	3
1	2	1	2	2	4	1	4	1	2
1	3	2	2	2	4	1	9	3	3
1	3	1	1	1	4	1	4	1	1
1	3	2	2	2	4	1	9	3	2
1	2	1	2	2	3	4	1	3	3
0	2	1	2	1	4	1	6	1	1
0	2	1	2	2	3	4	7	2	2
0	3	2	1	2	3	1	8	1	1
1	3	1	2	3	3	2	1	2	1
1	3	1	1	2	4	1	8	1	1
0	3	1	2	2	2	3	1	3	2
1	3	1	2	1	4	1	6	1	2
0	2	2	2	2	4	1	6	3	2
0	2	1	2	2	4	1	1	3	1
1	3	1	1	2	4	3	1	3	1

1	3	1	2	2	4	1	1	3	3
0	2	1	2	2	4	1	1	2	3
1	3	1	1	3	4	1	6	1	1
1	1	1	1	1	4	1	6	1	1
1	3	2	2	3	4	2	8	3	3
1	3	2	3	2	4	1	8	1	3
1	3	1	1	2	4	1	8	1	1
1	3	1	1	3	4	1	8	1	2
0	3	1	2	1	4	1	1	3	3
0	3	1	2	2	4	1	9	1	3
0	2	2	2	2	4	2	4	3	2
1	3	1	2	3	4	1	1	3	3
1	1	1	1	1	4	1	7	3	3
0	3	1	2	2	4	1	9	3	2
0	2	1	2	1	2	6	1	3	3
1	3	1	2	2	4	1	1	3	2
0	2	1	2	2	3	2	1	1	3
1	3	1	2	3	4	1	1	1	1
0	2	1	2	2	4	2	7	1	3
0	2	2	1	2	4	1	1	2	3
1	3	2	2	2	2	1	1	1	3
1	3	1	2	2	4	1	4	1	1
1	3	2	2	2	4	1	8	1	3
0	2	2	2	1	4	1	7	1	3
0	2	2	2	2	4	3	1	1	3
1	3	1	2	2	4	3	4	3	2
1	2	2	2	2	4	4	4	2	2
1	2	2	2	2	4	1	1	3	1
1	3	1	3	2	2	4	6	1	3
1	3	2	2	2	3	1	7	1	2
0	3	1	2	2	4	2	1	3	3
0	3	1	3	3	4	1	9	1	1
1	3	1	2	3	3	1	2	3	3
1	3	1	2	3	4	4	1	3	1
1	3	1	2	3	2	1	1	3	1
1	3	1	2	2	3	1	1	1	1
1	3	1	1	2	4	2	6	1	3
1	2	1	2	3	4	4	1	1	3
1	1	1	1	2	4	1	7	2	1
1	3	1	3	2	4	1	1	3	1
1	2	1	2	2	4	2	9	1	1
1	2	1	2	2	4	3	1	3	3
1	2	1	2	2	2	4	4	1	2
0	3	1	3	3	3	1	4	1	2
0	2	1	2	2	2	1	6	1	3
0	2	1	2	2	1	1	4	3	2
0	2	1	1	2	4	1	8	1	1

1	3	1	3	2	4	1	1	3	1
1	2	1	2	2	4	1	8	3	1
1	2	1	1	2	1	4	1	3	3
0	2	1	1	2	3	1	6	1	1
1	3	2	2	2	4	1	1	3	3
0	3	1	2	2	4	1	1	3	2
0	1	1	1	2	4	2	4	1	1
0	2	1	2	1	4	3	1	2	3
1	3	1	2	3	4	2	1	3	3
1	3	1	2	3	3	1	4	3	1
1	3	1	2	3	4	2	1	1	1
1	2	1	3	2	4	2	8	1	1
1	3	1	2	2	4	1	8	1	3
1	3	1	3	2	4	3	1	1	1
0	3	1	2	3	4	1	6	1	3
1	3	1	2	2	4	1	9	3	3
1	3	1	2	3	4	1	1	3	2
0	3	1	1	2	4	1	9	1	2
1	3	1	2	2	4	2	8	1	2
1	3	1	3	3	2	4	1	2	2
1	3	1	2	2	4	3	1	2	2
0	2	2	2	2	4	1	9	1	2
0	1	2	1	2	4	2	7	2	2
1	2	1	2	2	3	3	3	3	3
1	3	1	1	2	4	1	1	1	1
0	3	1	2	3	4	1	1	1	3
1	3	1	1	2	3	1	9	1	2
0	3	1	1	1	4	1	8	1	3
0	3	2	2	2	4	2	7	3	1
1	2	1	2	2	2	1	4	3	2
1	2	1	2	2	4	1	7	3	3
1	3	1	2	2	4	3	1	3	1
0	1	1	1	1	1	1	9	2	1
0	3	1	2	2	4	1	7	3	3
0	2	2	2	2	3	1	1	3	3
1	2	2	2	2	4	1	2	3	3
0	2	2	3	2	4	4	6	1	2
1	3	1	2	2	2	1	3	3	3
1	2	1	3	3	2	3	6	1	2
0	3	1	3	2	2	2	9	3	3
1	2	2	2	2	3	2	9	3	1
0	3	1	2	2	3	1	8	2	3
1	3	2	2	2	3	1	8	1	1
1	2	1	2	2	4	4	8	1	3
0	2	2	2	2	3	1	4	3	3
0	3	1	1	3	4	3	4	3	1
0	2	2	2	2	4	4	4	3	3

1	3	1	2	2	4	3	1	1	3
1	3	1	3	3	4	3	8	2	3
0	3	1	1	3	2	1	7	3	1
0	3	2	2	2	4	1	9	3	2
1	3	1	2	2	4	1	4	2	1
1	2	1	2	2	1	1	2	1	3
0	2	2	3	2	4	1	8	1	3
1	2	1	3	2	4	3	6	1	1
1	2	2	2	2	4	1	1	3	1
1	1	2	1	2	4	1	8	1	1
0	2	2	1	3	4	4	7	2	1
1	1	2	2	2	4	2	8	1	2
0	3	1	2	2	4	1	1	3	1
0	3	2	3	2	4	1	6	2	3
1	1	1	1	2	4	2	4	3	3
1	3	2	2	2	3	2	3	3	3
0	3	1	2	2	3	1	8	1	3
1	3	2	2	2	3	3	8	3	2
1	2	2	1	2	4	1	1	3	1
0	2	1	3	2	3	1	5	3	3
1	3	1	1	3	2	1	4	3	1
0	2	2	2	2	2	1	3	3	2
1	3	1	2	2	3	3	7	3	1
0	2	1	2	2	4	5	1	3	2
0	2	2	2	2	4	1	3	3	1
1	3	1	2	2	4	1	9	2	3
1	3	2	1	3	3	1	8	2	1
1	3	1	2	3	4	1	6	1	1
1	3	2	2	2	4	4	1	3	2
1	2	1	2	2	3	2	8	1	2
1	2	1	2	2	4	4	5	3	3
1	2	2	2	1	4	1	4	2	3
1	2	2	2	2	2	1	9	1	3
1	3	1	1	2	2	1	1	1	1
1	1	1	1	1	4	5	3	3	1
1	3	1	3	2	4	4	1	3	1
0	3	2	2	2	3	1	8	1	3
1	3	2	2	2	4	1	1	3	3
0	2	2	2	2	4	1	8	2	3
0	2	1	2	2	4	5	8	1	3
0	3	2	2	2	4	1	1	3	3
1	3	2	2	2	4	3	6	2	3
1	3	1	3	3	3	3	6	2	1
1	3	2	2	2	4	1	6	1	1
1	2	1	3	3	3	1	6	1	1
1	2	1	2	2	4	1	8	1	1
1	2	1	2	2	4	1	6	1	1

1	3	1	2	3	4	3	1	3	3
0	3	1	3	3	4	3	1	3	1
1	2	1	2	3	4	1	6	1	2
1	3	2	2	3	4	2	9	3	2
0	2	1	2	2	4	1	7	3	3
0	3	1	2	2	4	1	8	1	2
1	2	1	2	2	3	3	7	3	3
0	3	2	2	2	4	1	6	2	3
0	3	1	2	2	4	2	8	2	2
1	1	1	1	2	4	1	9	3	1
0	1	1	1	1	4	1	4	1	1
0	2	1	2	2	3	5	1	1	2
1	2	1	2	2	4	3	2	1	3
1	2	1	2	2	4	5	3	1	3
1	2	1	2	3	3	2	1	3	2
1	2	1	2	2	3	1	4	3	3
1	2	1	2	2	3	3	6	1	3
1	2	1	2	2	3	4	5	3	3
0	2	1	2	2	3	1	8	1	2
1	2	1	2	2	4	3	7	3	3
1	2	1	2	2	4	3	5	3	3
1	2	1	3	3	4	5	1	3	3
0	2	1	2	3	4	4	1	3	3
1	2	1	2	2	4	6	9	1	3
0	2	2	2	2	4	1	2	2	2
1	2	1	2	2	4	5	1	1	3
1	2	1	2	2	3	3	3	3	3
1	3	1	3	2	3	5	6	1	3
1	2	1	3	2	4	4	5	1	3
0	2	1	2	1	4	2	1	2	3
0	3	1	3	2	4	6	4	3	3
0	3	1	3	2	4	1	7	3	3
1	3	1	2	2	4	2	8	1	3
1	3	2	2	2	4	4	9	3	3
0	3	1	2	2	4	1	9	1	3
1	3	1	3	2	4	4	6	1	3
1	3	1	3	2	4	6	4	1	3
1	3	1	2	1	4	4	4	1	3
0	3	1	1	1	4	4	1	1	3
1	3	2	2	2	4	5	1	1	3
1	3	1	3	2	4	2	8	1	1
1	3	1	2	1	4	2	9	3	3
0	3	1	2	2	4	3	6	1	3
1	3	1	2	2	4	3	9	2	3
0	3	1	1	1	4	1	9	1	2
0	2	1	3	2	4	5	4	3	3
1	2	1	2	2	3	1	9	2	3

1	2	1	2	2	4	4	4	1	3
1	3	1	3	2	4	6	6	1	3
1	3	1	3	2	4	4	4	1	3
0	3	1	2	2	4	2	9	1	3
1	3	1	2	2	4	4	1	1	3
1	3	1	3	2	4	5	6	1	3
1	3	1	3	2	4	5	4	1	2
1	1	1	1	2	4	1	9	3	1
1	1	1	1	1	4	1	4	1	1
0	2	1	2	2	3	5	1	1	2
1	2	1	2	2	4	3	2	1	3
1	2	1	2	2	3	5	3	1	3
1	2	1	2	3	3	2	1	3	2
1	2	1	2	2	3	1	4	3	3
1	2	1	2	2	3	3	6	1	3
1	2	1	2	1	4	4	5	3	3
0	2	1	2	2	3	1	8	1	2
1	2	1	2	2	4	3	7	3	3
1	2	1	2	2	4	3	5	3	3
1	2	1	3	3	4	5	1	3	3
0	2	1	2	3	4	4	1	3	3
1	2	1	2	2	4	6	9	1	3
0	2	2	2	2	4	1	2	2	2
1	2	1	2	2	4	5	1	1	3
1	2	1	2	2	3	3	3	3	3
1	3	1	3	2	3	5	6	1	3
1	2	1	3	2	4	4	5	1	3
0	2	1	2	1	4	2	1	2	3
0	3	1	3	2	4	6	4	3	3
0	3	1	3	2	4	1	7	3	3
1	3	1	2	1	4	2	8	1	3
1	3	2	2	2	4	4	9	3	3
0	3	1	2	2	4	1	9	1	3
1	3	1	3	2	4	4	6	1	3
1	3	1	3	2	4	6	4	1	3
1	3	1	2	1	4	4	4	1	3
0	3	1	1	1	4	4	1	1	3
1	3	2	2	2	4	5	1	1	3
1	3	1	3	2	4	2	8	1	1
1	3	1	2	1	4	2	9	3	3
0	3	1	2	2	4	3	6	1	3
1	3	1	2	2	4	3	9	2	3
0	3	1	1	1	4	1	9	1	2
0	2	1	3	2	4	5	4	3	3
1	2	1	2	2	3	1	9	2	3
0	2	1	2	2	4	4	4	1	3
0	3	1	3	2	4	6	6	1	3

1	3	1	3	2	4	4	4	1	3
0	3	1	2	2	4	2	9	1	3
1	3	1	2	2	4	4	6	2	3
1	3	1	3	2	4	4	6	1	3
0	3	1	3	2	4	5	4	1	2
0	3	1	2	2	4	4	6	3	3
1	2	2	2	2	4	1	9	3	3
0	2	2	2	2	1	1	6	1	3
1	3	2	2	2	2	3	3	1	3
1	3	2	2	2	3	1	7	1	1
1	3	2	2	1	4	3	9	1	3
1	2	2	2	2	4	3	9	3	3
0	2	2	2	2	4	2	5	3	1
1	3	2	3	2	4	4	9	3	3
1	2	2	2	1	4	2	3	2	3
1	2	1	2	1	4	2	4	3	1
0	3	1	3	2	4	1	6	1	3
1	2	1	2	2	4	1	1	1	2
0	2	1	3	2	4	3	9	3	2
0	2	2	2	3	4	1	6	1	1
1	2	2	2	2	4	1	7	3	3
1	2	1	1	1	4	3	8	1	1
0	3	1	2	2	4	2	1	1	3
1	2	1	3	2	1	1	6	1	1
0	3	1	2	1	4	2	6	1	3
0	3	1	3	2	1	2	9	3	3
0	3	1	3	2	4	3	1	3	3
1	3	1	2	3	4	1	9	2	3
1	2	1	2	2	1	2	1	3	3
1	2	1	2	2	4	2	4	1	2
1	3	2	2	2	4	1	9	3	3
1	3	1	1	1	4	2	4	1	1
1	2	1	2	2	3	4	1	3	3
0	2	1	2	1	4	2	6	1	1
0	2	1	2	2	3	4	7	2	2
0	3	2	1	2	3	4	8	1	1
1	3	1	2	3	3	2	1	2	1
1	3	1	1	2	4	3	1	3	2
0	3	1	2	1	4	3	6	1	2
0	2	2	2	2	4	1	6	3	2
1	3	1	1	2	4	3	1	3	1
1	3	1	2	2	4	3	1	3	3
0	2	1	2	2	4	2	1	1	3
1	3	1	2	3	4	2	6	1	1
1	1	1	1	1	4	3	6	1	1
1	3	2	2	3	4	2	8	1	3
1	3	2	3	2	4	2	8	1	3

1	3	1	1	2	4	1	8	1	1
0	3	1	2	1	4	2	1	3	3
0	3	1	2	2	4	1	9	1	3
0	2	2	2	2	4	2	4	3	2
0	2	1	2	1	2	6	1	3	3
0	2	2	2	2	3	2	1	1	3
0	2	1	2	2	4	2	7	1	3
0	2	2	1	2	4	2	1	2	3
1	3	2	2	2	2	1	1	1	3
1	3	2	2	2	4	1	8	1	3
0	2	2	2	2	4	3	1	1	3
1	3	1	2	2	4	3	4	3	2
1	2	2	2	2	4	4	4	2	2
1	3	1	3	2	2	4	6	1	3
0	3	1	2	2	4	3	1	3	3
0	3	1	3	3	4	1	9	1	1
1	3	1	2	3	4	4	1	3	1
1	3	1	2	3	2	3	1	3	1
1	3	1	2	2	3	3	1	1	1
0	3	1	1	2	4	3	6	1	3
1	2	1	2	3	4	4	1	1	3
1	3	1	3	2	4	4	1	3	1
1	2	1	2	2	4	3	9	1	1
0	2	1	2	2	4	4	1	3	3
1	2	1	2	2	2	4	1	1	2
0	2	1	2	2	2	3	6	1	3
1	2	1	1	2	1	4	1	3	3
0	2	1	1	2	3	1	6	1	1
1	3	2	2	2	4	3	1	3	3
1	1	1	1	2	4	3	4	1	1
0	2	1	2	1	4	3	1	2	3
1	3	1	2	3	4	3	1	1	3
1	3	1	2	2	4	3	8	1	3
0	3	1	3	2	4	3	1	1	1
0	3	1	2	3	4	1	6	1	3
1	3	1	3	3	2	4	1	2	2
1	3	1	2	2	4	3	1	2	2
1	3	1	1	2	4	3	1	1	1
0	3	1	1	1	4	1	8	1	3
0	2	2	3	2	4	4	6	1	2
0	2	1	3	3	2	3	6	1	2
1	2	2	2	2	3	3	9	3	1
0	2	1	2	2	4	4	8	1	3
1	3	1	3	3	4	3	8	2	3
0	2	2	3	2	4	2	8	1	3
1	2	1	3	2	4	4	6	1	1
0	2	2	1	3	4	4	7	2	1

1	1	2	2	2	4	2	8	1	2
1	3	2	2	2	3	2	3	3	3
0	3	1	2	2	3	1	8	1	3
0	2	1	2	2	4	5	1	3	2
1	3	2	2	2	4	4	1	3	2
1	2	1	2	2	4	4	5	3	3
1	2	2	2	1	4	1	4	2	3
1	1	1	1	1	4	5	3	3	1
1	3	1	3	2	4	4	1	3	1
0	3	2	2	2	3	1	8	1	3
1	2	2	2	2	4	1	8	2	3
0	2	1	2	2	4	5	8	1	3
1	3	1	3	3	3	3	6	2	1
1	3	2	2	2	4	4	6	1	1
1	3	1	2	3	4	3	1	1	3
1	2	1	2	2	4	4	7	3	3

LSMVcat1	_st	_d	_t	_t0	RRcat	DBpcat	CMVcat
2	1	1	14	0	3	2	2
2	1	1	14	0	3	2	2
2	1	1	10	0	3	2	1
3	1	1	39	0	3	2	3
1	1	1	4	0	3	2	1
3	1	1	13	0	2	2	2
3	1	1	10	0	3	2	6
1	1	1	3	0	2	1	6
3	1	1	32	0	3	1	3
3	1	1	36	0	3	2	4
1	1	1	3	0	2	2	1
3	1	1	14	0	3	2	3
2	1	1	7	0	2	2	5
3	1	1	13	0	2	2	1
1	1	1	9	0	3	2	1
1	1	1	9	0	3	3	6
1	1	1	6	0	3	2	1
1	1	1	4	0	3	2	2
2	1	1	9	0	3	2	1
2	1	1	5	0	3	2	2
1	1	1	5	0	3	2	1
3	1	1	46	0	2	2	1
1	1	1	3	0	2	1	1
3	1	1	35	0	3	1	1
1	1	1	3	0	3	3	5
3	1	1	22	0	1	2	2
3	1	1	29	0	3	2	1
3	1	1	16	0	3	2	1
3	1	1	13	0	3	2	5
3	1	1	57	0	3	2	1
3	1	1	27	0	3	2	2
2	1	1	4	0	3	2	2
3	1	1	29	0	2	2	5
1	1	1	1	0	1	1	2
1	1	1	4	0	2	1	3
3	1	1	26	0	3	2	1
1	1	1	1	0	1	2	2
2	1	1	20	0	3	2	1
1	1	1	1	0	3	2	1
1	1	1	1	0	3	1	1
1	1	1	1	0	3	1	1
1	1	1	4	0	3	3	1
2	1	1	6	0	3	2	1
2	1	1	15	0	3	2	3
1	1	1	3	0	3	2	1
1	1	1	1	0	3	1	1

3	1	1	16	0	3	1	1
3	1	1	18	0	3	2	1
1	1	1	1	0	3	2	1
1	1	1	3	0	3	2	1
3	1	1	30	0	3	2	3
3	1	1	70	0	3	3	6
1	1	1	3	0	3	1	2
2	1	1	4	0	3	1	1
2	1	1	11	0	3	3	1
2	1	1	10	0	3	2	1
2	1	1	6	0	2	2	2
3	1	1	12	0	3	2	1
2	1	1	12	0	2	1	1
1	1	1	7	0	3	2	6
3	1	1	12	0	3	2	1
2	1	1	8	0	3	2	3
2	1	1	9	0	2	2	2
1	1	1	2	0	3	2	2
3	1	1	24	0	2	2	1
3	1	1	20	0	3	1	2
1	1	1	10	0	3	2	3
1	1	1	6	0	3	1	2
3	1	1	18	0	3	3	3
3	1	1	13	0	3	1	4
2	1	1	27	0	3	2	3
2	1	1	28	0	3	2	1
2	1	1	4	0	3	2	1
1	1	1	3	0	3	2	3
3	1	1	34	0	3	3	1
2	1	1	6	0	3	2	1
3	1	1	15	0	3	3	1
1	1	1	2	0	3	2	2
2	1	1	11	0	3	2	1
1	1	1	2	0	3	2	1
1	1	1	5	0	2	2	1
1	1	1	3	0	3	1	1
3	1	1	28	0	3	1	2
3	1	1	37	0	2	1	2
1	1	1	1	0	2	2	1
1	1	1	1	0	3	3	1
1	1	1	1	0	3	1	1
3	1	1	29	0	2	2	1
2	1	1	9	0	3	1	1
1	1	1	4	0	3	3	1
1	1	1	9	0	3	2	2
2	1	1	5	0	3	2	1
1	1	1	3	0	2	1	1

1	1	1	15	0	3	2	1
1	1	1	11	0	2	2	1
2	1	1	16	0	3	1	1
1	1	1	3	0	2	2	2
3	1	1	13	0	3	2	1
2	1	1	6	0	3	3	1
1	1	1	2	0	3	2	1
3	1	1	10	0	3	2	1
3	1	1	18	0	3	2	1
1	1	1	1	0	3	2	1
1	1	1	3	0	3	2	1
1	1	1	3	0	3	2	1
3	1	1	15	0	3	2	1
1	1	1	1	0	3	3	1
3	1	1	10	0	3	1	2
3	1	1	10	0	3	2	3
2	1	1	6	0	3	2	1
1	1	1	7	0	3	1	1
2	1	1	5	0	3	2	2
2	1	1	4	0	3	2	1
2	1	1	7	0	3	2	1
1	1	1	6	0	3	2	2
2	1	1	9	0	3	1	1
3	1	1	18	0	2	2	3
1	1	1	3	0	3	2	1
3	1	1	15	0	3	2	1
2	1	1	6	0	3	1	5
3	1	1	31	0	3	1	1
1	1	1	3	0	3	2	1
2	1	1	4	0	2	2	1
3	1	1	15	0	1	2	1
1	1	1	3	0	3	2	1
1	1	1	5	0	2	2	5
3	1	1	21	0	3	2	1
1	1	1	26	0	3	2	1
3	1	1	27	0	2	2	1
2	1	1	5	0	3	3	2
2	1	1	8	0	2	2	1
2	1	1	5	0	3	3	2
3	1	1	10	0	3	3	1
1	1	1	4	0	2	2	5
1	1	1	9	0	2	2	1
1	1	1	2	0	3	1	1
3	1	1	14	0	2	2	1
3	1	1	8	0	3	3	4
1	1	1	1	0	3	2	1
1	1	1	15	0	2	2	4

3	1	1	13	0	1	1	1
3	1	1	49	0	3	2	1
1	1	1	3	0	3	2	1
2	1	1	6	0	2	1	1
1	1	1	2	0	3	2	1
3	1	1	13	0	2	2	5
3	1	1	28	0	2	3	1
1	1	1	3	0	3	3	1
1	1	1	1	0	2	2	1
1	1	1	2	0	3	1	1
1	1	1	14	0	3	2	1
2	1	1	5	0	3	2	2
1	1	1	2	0	3	2	1
3	1	1	42	0	3	2	1
3	1	1	12	0	3	2	1
3	1	1	29	0	3	2	3
3	1	1	10	0	3	2	1
2	1	1	6	0	3	2	1
1	1	1	3	0	2	1	1
3	1	1	14	0	3	3	4
1	1	1	1	0	3	1	1
2	1	1	11	0	3	2	2
1	1	1	1	0	3	2	1
1	1	1	4	0	3	2	1
1	1	1	3	0	3	2	1
3	1	1	20	0	3	2	1
1	1	1	1	0	3	1	1
1	1	1	12	0	3	1	2
2	1	1	5	0	3	2	4
2	1	1	11	0	3	2	1
1	1	1	8	0	3	1	1
3	1	1	24	0	2	2	1
3	1	1	9	0	3	2	1
1	1	1	1	0	3	1	1
1	1	1	2	0	3	2	1
1	1	1	3	0	3	3	1
3	1	1	41	0	3	2	1
3	1	1	26	0	3	2	1
3	1	1	27	0	3	2	1
3	1	1	28	0	3	2	1
3	1	1	20	0	3	2	1
3	1	1	17	0	2	2	1
1	1	1	1	0	2	3	1
1	1	1	1	0	2	2	5
1	1	1	2	0	3	2	2
1	1	1	1	0	3	2	1
1	1	1	1	0	3	2	2

3	1	1	19	0	3	2	1
1	1	1	2	0	3	3	1
2	1	1	6	0	1	2	2
2	1	1	8	0	3	2	1
3	1	1	29	0	2	2	1
2	1	1	16	0	2	2	1
3	1	1	11	0	3	2	1
3	1	1	27	0	3	2	2
1	1	1	5	0	3	2	1
1	1	1	1	0	3	2	1
1	1	1	1	0	3	2	2
2	1	1	5	0	3	1	1
3	1	1	24	0	2	2	2
3	1	1	27	0	2	2	1
2	1	1	7	0	3	2	1
3	1	1	12	0	3	2	4
3	1	1	29	0	2	2	2
3	1	1	18	0	2	2	1
2	1	1	7	0	3	2	1
3	1	1	25	0	2	2	1
3	1	1	30	0	3	2	4
3	1	1	25	0	3	3	1
3	1	1	17	0	3	2	1
3	1	1	9	0	3	2	1
2	1	1	5	0	2	2	5
3	1	1	33	0	2	2	3
3	1	1	31	0	2	2	3
3	1	1	26	0	1	3	2
3	1	1	28	0	2	2	1
3	1	1	24	0	2	2	1
3	1	1	35	0	2	3	2
3	1	1	41	0	2	2	1
3	1	1	31	0	3	2	1
3	1	1	32	0	2	2	3
3	1	1	15	0	3	2	2
3	1	1	23	0	1	2	2
3	1	1	18	0	2	3	2
3	1	1	9	0	2	2	1
3	1	1	25	0	2	1	1
3	1	1	43	0	2	2	2
1	1	1	3	0	3	3	1
3	1	1	30	0	3	2	3
3	1	1	29	0	2	2	2
3	1	1	26	0	2	2	3
1	1	1	5	0	2	1	1
2	1	1	8	0	2	3	1
3	1	1	24	0	2	2	2

3	1	1	22	0	3	2	1
3	1	1	25	0	1	3	2
3	1	1	27	0	2	3	2
3	1	1	36	0	2	3	3
3	1	1	25	0	2	2	1
3	1	1	28	0	2	3	2
2	1	1	4	0	2	3	2
1	1	1	1	0	3	2	1
1	1	1	1	0	3	2	2
2	1	1	5	0	3	1	1
3	1	1	24	0	2	2	2
3	1	1	27	0	2	2	1
2	1	1	7	0	3	2	1
3	1	1	12	0	3	2	4
3	1	1	29	0	2	2	2
3	1	1	18	0	2	2	1
2	1	1	7	0	3	2	1
3	1	1	25	0	2	2	1
3	1	1	30	0	3	2	4
3	1	1	25	0	3	3	1
3	1	1	17	0	3	2	1
3	1	1	9	0	3	2	1
2	1	1	5	0	2	2	5
3	1	1	33	0	2	2	3
3	1	1	31	0	2	2	3
3	1	1	26	0	1	3	2
3	1	1	28	0	2	2	1
3	1	1	24	0	2	2	1
3	1	1	35	0	2	3	2
3	1	1	41	0	2	2	1
3	1	1	31	0	3	2	1
3	1	1	32	0	2	2	3
3	1	1	15	0	3	2	2
3	1	1	23	0	1	2	2
3	1	1	18	0	2	3	2
3	1	1	9	0	2	2	1
3	1	1	25	0	2	1	1
3	1	1	43	0	2	2	2
1	1	1	3	0	3	3	1
3	1	1	30	0	3	2	3
3	1	1	29	0	2	2	2
3	1	1	26	0	2	2	3
1	1	1	5	0	2	1	1
2	1	1	8	0	2	3	1
3	1	1	24	0	2	2	2
3	1	1	22	0	3	2	1
3	1	1	25	0	1	3	2

3	1	1	27	0	2	3	2
3	1	1	36	0	2	3	3
3	1	1	25	0	2	2	1
3	1	1	28	0	2	3	2
2	1	1	4	0	2	3	2
2	1	1	14	0	3	2	2
3	1	1	39	0	3	2	3
3	1	1	13	0	2	2	2
3	1	1	10	0	3	2	6
1	1	1	3	0	2	1	6
3	1	1	32	0	3	1	3
3	1	1	36	0	3	2	4
1	1	1	3	0	2	2	1
3	1	1	14	0	3	2	3
3	1	1	13	0	2	2	1
1	1	1	4	0	3	2	1
1	1	1	9	0	3	3	1
1	1	1	4	0	3	2	2
2	1	1	9	0	3	2	1
1	1	1	5	0	3	2	1
3	1	1	59	0	2	2	1
1	1	1	3	0	2	1	1
3	1	1	35	0	3	1	1
1	1	1	3	0	3	3	5
3	1	1	22	0	1	2	2
3	1	1	29	0	3	2	1
3	1	1	16	0	3	2	1
3	1	1	13	0	3	2	5
3	1	1	27	0	3	2	2
2	1	1	4	0	3	2	2
3	1	1	29	0	2	2	5
1	1	1	1	0	1	1	2
3	1	1	26	0	3	2	1
1	1	1	1	0	1	2	2
2	1	1	20	0	3	2	1
1	1	1	1	0	3	2	1
1	1	1	1	0	3	1	1
1	1	1	4	0	3	1	1
2	1	1	6	0	3	2	1
2	1	1	15	0	3	2	3
1	1	1	1	0	3	1	1
3	1	1	16	0	3	1	1
3	1	1	18	0	3	2	1
1	1	1	1	0	3	2	1
1	1	1	3	0	3	2	1
3	1	1	30	0	3	2	3
3	1	1	70	0	3	3	6

1	1	1	3	0	3	1	2
2	1	1	11	0	3	3	1
2	1	1	10	0	3	2	1
2	1	1	6	0	2	2	2
3	1	1	12	0	3	2	1
2	1	1	9	0	2	2	2
3	1	1	24	0	2	2	1
3	1	1	20	0	3	1	2
3	1	1	10	0	3	2	3
3	1	1	18	0	3	3	3
2	1	1	27	0	3	2	3
2	1	1	28	0	3	2	1
2	1	1	4	0	3	2	1
3	1	1	34	0	3	3	1
3	1	1	15	0	3	3	1
1	1	1	2	0	3	2	2
1	1	1	2	0	3	2	1
1	1	1	5	0	2	2	1
1	1	1	3	0	3	1	1
3	1	1	28	0	3	1	2
3	1	1	37	0	2	1	2
1	1	1	1	0	3	3	1
1	1	1	1	0	3	1	1
3	1	1	29	0	2	2	1
2	1	1	9	0	3	1	1
1	1	1	9	0	3	2	2
3	1	1	19	0	3	1	1
1	1	1	3	0	2	2	2
3	1	1	13	0	3	2	1
1	1	1	2	0	3	2	1
3	1	1	10	0	3	2	1
3	1	1	18	0	3	2	1
3	1	1	15	0	3	2	1
1	1	1	1	0	3	3	1
3	1	1	10	0	3	1	2
2	1	1	4	0	3	2	1
2	1	1	7	0	3	2	1
1	1	1	3	0	3	2	1
3	1	1	6	0	3	1	1
2	1	1	10	0	3	3	2
2	1	1	10	0	3	3	2
1	1	1	5	0	2	2	5
3	1	1	14	0	2	2	1
3	1	1	49	0	3	2	1
3	1	1	28	0	2	3	1
1	1	1	3	0	3	3	1
1	1	1	14	0	3	2	1

2	1	1	5	0	3	2	2
3	1	1	29	0	3	2	3
3	1	1	10	0	3	2	1
1	1	1	4	0	3	2	1
2	1	1	5	0	3	2	1
1	1	1	8	0	3	1	1
3	1	1	24	0	2	2	1
1	1	1	2	0	3	2	1
1	1	1	3	0	3	3	1
3	1	1	41	0	3	2	1
3	1	1	27	0	3	2	1
3	1	1	28	0	3	2	1
1	1	1	1	0	2	3	1
1	1	1	1	0	2	2	5
3	1	1	19	0	3	2	1
3	1	1	29	0	2	2	1
